# Dietary Inflammatory Potential, Inflammation-Related Lifestyle Factors, and Incident Anxiety Disorders: A Prospective Cohort Study

**DOI:** 10.3390/nu16010121

**Published:** 2023-12-29

**Authors:** Jiali Zheng, Mengdan Liu, Longgang Zhao, James R. Hébert, Susan E. Steck, Hui Wang, Xiaoguang Li

**Affiliations:** 1 Department of Epidemiology and Biostatistics, School of Public Health, Shanghai Jiao Tong University School of Medicine, Shanghai 200025, China; jzheng@shsmu.edu.cn; 2 Department of Food Safety and Toxicology, School of Public Health, Shanghai Jiao Tong University School of Medicine, Shanghai 200025, China; mengdliu@sjtu.edu.cn; 3 Department of Epidemiology and Biostatistics, Arnold School of Public Health, University of South Carolina, Columbia, SC 29208, USA; lz7@email.sc.edu (L.Z.); jhebert@mailbox.sc.edu (J.R.H.); stecks@mailbox.sc.edu (S.E.S.); 4 Cancer Prevention and Control Program, Arnold School of Public Health, University of South Carolina, Columbia, SC 29208, USA

**Keywords:** dietary inflammatory potential, inflammation-related lifestyle factors, anxiety disorders, prospective cohort study

## Abstract

It is unclear whether diet-associated inflammation is related to the development of anxiety disorders. We aimed to investigate the association between energy-adjusted dietary inflammatory index (E-DII) scores and the incidence of anxiety disorders, and explore the joint effects of E-DII scores with other inflammatory lifestyles in enhancing anxiety risk. In the UK Biobank Study of 96,679 participants, baseline E-DII scores were calculated from the average intake of at least two 24 h dietary recalls. Multivariable-adjusted Cox models were used to evaluate the associations between E-DII scores and the incidence of total anxiety disorders, and primary types and subtypes; additive and multiplicative interactions of a pro-inflammatory diet and seven inflammatory lifestyles were examined. After a median follow-up of 9.4 years, 2785 incident cases of anxiety disorders occurred. Consuming a pro-inflammatory diet was significantly associated with a higher risk of total anxiety disorders (HR_Q4vsQ1_ = 1.12, 95% CI = 1.00–1.25), and positive associations were consistently identified for primary types and subtypes of anxiety disorders, with HRs ranging from 1.08 to 1.52, and were present in women only. Both additive and multiplicative interactions of current smoking and a proinflammatory diet on total anxiety risk were identified. A proinflammatory diet was associated with a higher incidence of anxiety disorders, and current smoking may synergize with a proinflammatory diet to promote anxiety risk, particularly among women.

## 1. Introduction

Anxiety disorders, which include main types such as phobic anxiety disorders, generalized anxiety disorder (GAD), panic disorders, and obsessive-compulsive disorder (OCD), are the most common mental illness in the world and follow a chronic course in one’s life [1,2]. They are associated with significant functional impairment, an increased likelihood of comorbidities, as well as high health-care utilization [2]. The etiology of anxiety is not fully clear, although personality, genetic factors, and environmental stressors are proposed as contributing factors [3], thus implying the necessity of identifying modifiable risk factors for effective prevention and intervention to reduce its substantial burden.

The emerging field of nutritional psychiatry provides mechanistic, observational, and interventional evidence to suggest that diet quality may be a modifiable risk factor for mental illnesses [4,5]. Summary evidence from clinical trials and cohort studies indicates that a healthy dietary pattern is associated with lower depression risk. However, research on anxiety has so far lagged behind that of depression, and findings have been less consistent mainly due to methodological issues, including populations under study with or without pre-existing medical conditions and inconsistent definitions of symptom-based anxiety rather than using standard clinically diagnosed anxiety disorders as a study outcome [5,6].

Diet may directly or indirectly influence anxiety development through several mechanisms, for which chronic inflammation serves as a common substrate, such as those involved in the production and metabolism of neurotransmitters and the microbiome-gut-brain axis (MGBA) [7,8,9]. To date, with the use of the dietary inflammatory index (DII^®^), several studies have evaluated the inflammatory potential of the diet in association with anxiety symptoms, generating inconsistent findings [10,11,12,13,14,15,16,17,18,19]. All these studies have been cross-sectional or case-control in design, limiting inferences about causality, especially when the association between diet and mental disorders is complex and likely to be bidirectional [5]. Prospective studies with long follow-ups are essential to accurately evaluate the diet and mental health relationship. To our knowledge, there has been no prospective study investigating how dietary inflammatory potential may impact the development of clinically diagnosed anxiety risk.

Therefore, to address this research gap, we aimed to examine the prospective association between dietary inflammatory potential, as assessed with the energy-adjusted DII (E-DII^TM^) score [20], and incident anxiety disorders, as well as primary types and subtypes in the UK Biobank study, and further investigate whether there were joint effects of E-DII scores and other inflammation-related lifestyle factors in enhancing incident anxiety disorders [21], including body mass index (BMI), cigarette smoking, alcohol drinking, sleep quality, physical activity, nonsteroidal anti-inflammatory drug (NSAID) use, and vitamin/mineral supplement use.

## 2. Materials and Methods

### 2.1. Study Design and Study Population

The UK Biobank study is a large prospective cohort study with the population and study design described in detail previously [22]. Briefly, more than 500,000 participants aged 40–70 years from the general population were recruited from 22 assessment centers throughout the United Kingdom between 2006 and 2010. Each eligible participant completed a written informed consent form and provided sociodemographic, lifestyle, and other health-related information through touchscreen questionnaires, verbal interviews, physical measurements, and biological sample collection at a baseline assessment center visit [22].

**Figure 1:**
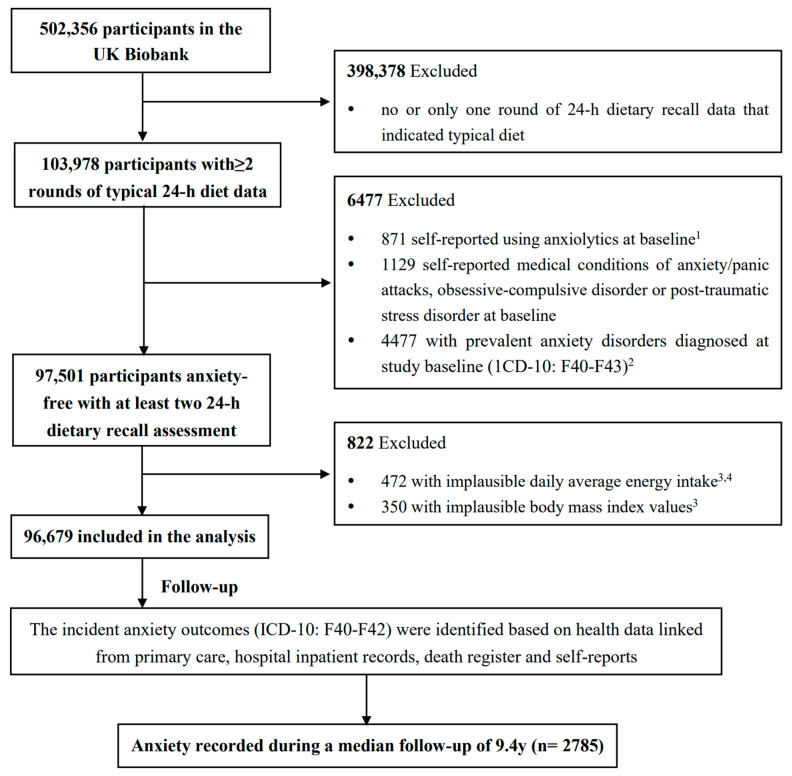
**The flowchart of study participant selection in the UK Biobank.** ^1^ Anxiolytics list was noted in Supplementary Information S1. ^2^ Prevalent anxiety disorders at baseline were determined by using the “first occurrence” diagnosis date mapped to ICD-10 codes of F40–F43 (Fields 130905, 130907, 130909, 130911). ^3^ Implausible daily average energy intake or body mass index were defined as 2 interquartile ranges above the sex-specific 75th percentile or below the 25th percentile of log-transformed variables. ^4^ Daily average energy intake was calculated by averaging the daily energy intake values from all the included rounds of 24 h dietary recalls.

In our analyses, participants were excluded if they (1) completed less than two rounds of 24 h dietary recalls with a typical diet indicated (n = 398,378); (2) had prevalent anxiety disorders at study baseline, including those who self-reported using anxiolytics (n = 871), or having a medical history of an anxiety disorder (n = 1129), or had ever received anxiety diagnosis mapped to ICD codes of F40–F43 (F40: phobic anxiety disorders; F41: other anxiety disorders; F42: OCD; F43: reaction to severe stress, and adjustment disorders) which was linked from hospital inpatient records, primary care, or self-report in the UK Biobank at or before baseline (n = 4477); (3) reported an extreme daily average energy intake or BMI (i.e., 2 interquartile ranges below the sex-specific 25th percentile or above the 75th percentile of log-transformed daily average energy intake or BMI, n = 472 and 350, respectively). Finally, a total of 96,679 participants were included in the analysis. The selection procedure of baseline study participants was described in Figure 1. The UK Biobank has approval from the North West Multi-center Research Ethics Committee. The present study was conducted under application number 83,432 of the UK Biobank resource.

### 2.2. Dietary Assessment (2009–2012)

In the UK Biobank, a web-based self-administered 24 h dietary recall questionnaire, the Oxford WebQ, was used to seek participants’ dietary intake information about up to 206 foods and 32 beverages over the previous day [23]. It was completed by 14% of participants in 2009–2010 for the first occasion. Subsequently, between 2011 and 2012, participants with valid email addresses (~330,000 participants) were invited to complete the Oxford WebQ on four separate occasions, administered every 3–4 months apart and on variable days of the week. On the questionnaire, participants were asked to indicate if what they ate and drank yesterday was typical of their usual consumption. The quantity of a food or beverage item was calculated by multiplying the assigned portion size of each item by the self-reported number of portions consumed [24]. Total energy and nutrient intakes were calculated by multiplying the food or beverage quantity with its nutrient composition from the UK Nutrient Databank [24]. The Oxford WebQ has been validated against an interviewer-administered 24 h dietary recall and recovery biomarkers, and found to have good performance [23,25]. To improve the representativeness of habitual diet, the current analysis was limited to participants who completed at least two WebQs, with each indicating a typical diet among the total five occasions. We calculated and used the average daily dietary intake amount for each individual as the mean value from all the included WebQs.

### 2.3. Calculation of E-DII Score

The E-DII score was calculated by linking the individual daily average intake with literature-derived inflammatory effect scores for each food parameter included in the DII [26]. The details of the development of DII have been published previously [26]. Briefly, inflammatory effect scores for 45 food parameters (i.e., components of DII), including macronutrients, micronutrients, some foods, and bioactive components, were derived based on qualifying research articles published until 2010 on the effect of dietary factors on six well-established inflammatory biomarkers (Interleukin (IL)-1β, IL-4, IL-6, IL-10, tumor necrosis factor (TNF)-α, and C-reactive protein (CRP)) [26]. The daily average food and nutrient consumption, calculated from WebQs, were first adjusted for total energy using a nutrient-density approach. The energy-adjusted dietary intake was subsequently standardized to a worldwide dietary database representing energy-adjusted dietary intake from 11 worldwide populations to avoid arbitrariness by simply using raw intake amounts. Then, the energy-adjusted standardized dietary intake was multiplied by the inflammatory effect score for each DII component and summed across all components to obtain the overall E-DII score [26]. Higher E-DII scores represent more proinflammatory diets, while lower (i.e., more negative) E-DII scores indicate more anti-inflammatory diets. The DII score has been construct-validated against inflammatory biomarkers in over 40 populations and found to be associated with higher levels of IL-6, TNF-α receptor 2, and high-sensitivity CRP [27,28].

In this study, 32 out of the 45 food parameters were used to calculate the E-DII score due to data availability, including energy, carbohydrate, protein, total fat, alcohol, fiber, cholesterol, saturated fat, monounsaturated fat, polyunsaturated fat, n-3 fatty acids, n-6 fatty acids, trans fat, niacin, thiamin, riboflavin, vitamin B12, vitamin B6, iron, magnesium, zinc, selenium, vitamin A, vitamin C, vitamin D, vitamin E, folic acid, β-carotene, garlic, onion, pepper, and caffeine. Among these 32 food parameters, we manually calculated the quantity of garlic, onion, pepper, and total caffeine, while estimates of the intake of the other 28 food parameters were derived from the UK Biobank. Total caffeine consumption was calculated by adding the products of the consumption of each caffeine-containing food and respective caffeine content based on published caffeine content reports [29,30]. The E-DII score in this study was calculated from the diet only because the amount of supplement intake was unknown.

### 2.4. Assessment of Incident Outcomes of Anxiety Disorders

Anxiety disorders in this study included three primary types with corresponding ICD-10 codes—phobic anxiety disorders (F40), other anxiety disorders (F41), and OCD (F42) [31,32]. Other anxiety disorders (F41), the largest primary type of anxiety disorders in the UK Biobank accounting for 90.7% of all anxiety cases, included the following subtypes: panic disorders (F41.0), GAD (F41.1), mixed anxiety and depressive disorder (F41.2), other mixed anxiety disorders (F41.3), specified anxiety disorders (F41.8), and unspecified anxiety disorder (F41.9), among which unspecified anxiety disorder was the largest subtype in this study population, accounting for 65.5% of all anxiety cases. In the present study, we analyzed following anxiety outcomes: (1) total anxiety disorders (i.e., F40-F42), (2) phobic anxiety disorders, (3) other anxiety disorders, and (4) all the subtypes under “other anxiety disorders” that had adequate case numbers for analysis (i.e., F41.0, F41.2, F41.9). We did not analyze OCD due to very few number of cases identified (n = 16). Incident cases of an anxiety disorder were identified through multiple sources, including self-reported diagnosis, hospital inpatient records, primary care data, and death registry records. Diagnoses from these sources were all mapped to ICD-10 codes, and the related incidence information was integrated to generate the “first occurrence” data in the UK Biobank, which was used to define the diagnosis date of an incident anxiety disorder and the source where the disorder was first reported. As the incidence information was only provided for three primary types of anxiety disorders, we used hospital inpatient record to determine the first diagnosis for any subtype of anxiety disorders. Given that the pathogenesis and etiologies of the three primary types of anxiety disorders may vary, we treated these outcomes separately when a participant had multiple disorders [2].

### 2.5. Assessment of Other Covariates

Information on important sociodemographic factors and lifestyle factors, including cigarette smoking, alcohol drinking, sleep quality, physical activity, and nonsteroidal anti-inflammatory drugs (NSAIDs) use, was all collected through touchscreen questionnaires or verbal interviews at baseline [22]. Sleep quality was assessed with a healthy sleep score calculated by summing the number of low-risk sleep factors based on responses to five sleeping-related questions [33]. Physical activity was assessed with Metabolic Equivalent Task (MET)-minutes per week for all activities, including walking, moderate, and vigorous activity. A vitamin/mineral supplement user was defined as an individual who had positive response to the question of whether or not they used supplement in more than half of their included 24 h dietary recalls [23]. BMI was calculated as weight(kg)/height(m)^2^, with weight and height measured at the baseline visit. In this analysis, we included cardiovascular diseases, hypertension, hyperlipidemia, type 2 diabetes mellitus, cancers, digestive diseases, and chronic kidney diseases as diet and anxiety-related comorbidities [34,35] based on self-reported diagnoses, medication use, and hospital admission data. Baseline depression status was defined by symptom-based outcomes derived from participants’ responses to mental health questions on the touchscreen questionnaire [36]. Details on the definitions and categorization of all the covariates are provided in the Supplementary Method section.

### 2.6. Statistical Analysis

The baseline characteristics of the study population were presented with means and standard deviations for continuous variables and numbers and frequencies for categorical variables by quartiles of E-DII scores. Analysis of Variance (ANOVA) test for continuous variables and Chi-square tests for categorical variables were used to test the differences across E-DII quartiles.

For each anxiety outcome, participants were followed up from baseline to the diagnosis of incident anxiety disorder, loss to follow-up, death, or the end of study follow-up (30 September 2021 for England and Wales, 31 October 2021 for Scotland), whichever came first. Cox proportional hazards models with person-years as the underlying time metric were used to estimate the age-, sex-, energy-adjusted, and multivariable-adjusted HRs and 95% confidence intervals (CIs) of an anxiety disorder, with the lowest E-DII quartile (the most anti-inflammatory diet) as the reference. The Schoenfeld residual test was performed to examine the proportional hazard (PH) assumption [37]. Given that only age as a covariate violated the PH assumption, we fitted an extended Cox model stratified by binary age group with median as the cutoff. The linear trend of anxiety risk across quartiles of E-DII scores was tested using a continuous E-DII score, after confirming the linear assumption was sufficient based on the restricted cubic spline test [38]. The E-DII score also was analyzed as a continuous variable, with the HR and 95% CI estimated for each SD increase of the E-DII score. In the multivariable-adjusted models, age groups (<58, ≥58), sex, total energy intake (kcal/day), ethnicity (Asian or Asian British, Black or Black British, Chinese, mixed, other ethnic groups, White, unknown), education qualification (college or university degree/vocational qualification, national examination at age 17–18, national examination at age 16, unknown), Townsend deprivation index (least deprived, intermediate, most deprived, unknown), cigarette smoking status (never, past smokers ≥ 15 cigarettes/d, past smokers < 15 cigarettes/d, past smokers with amount unknown, current smokers ≥ 15 cigarettes/d, current smokers < 15 cigarettes/d, current smokers with smoked amount unknown, unknown status), alcohol drinking status (never, past drinkers, current drinkers ≤ 7.1 g/d, current drinkers with 7.2–18.6 g/d, current drinkers > 18.6 g/d, unknown status), physical activity (low, medium, high, unknown), depression status (no, yes), sleep quality (healthy, intermediate, poor, unknown), BMI status (underweight, normal, overweight, obese), vitamin/mineral supplement use (no, yes), and the number of diet and anxiety-related comorbidities (0, 1–2, ≥3) were adjusted according to previous literature [34,39,40]. Given the potential sex difference in the risk of anxiety and dietary intake [2], we conducted analyses for men and women separately and combined. Stratified associations between E-DII score and total anxiety as well as primary types were also conducted by sociodemographic factors including age groups, education qualification, and Townsend deprivation index, and by the number of comorbidities.

In the joint effect analysis, we selected a priori seven inflammation-related lifestyle factors, including BMI, smoking status, alcohol drinking, sleep quality, physical activity, NSAIDs use, and vitamin/mineral supplement use based on previous evidence of the associations of these lifestyles with chronic inflammation [41,42,43]. The multivariable-adjusted associations between each inflammation-related lifestyle factor and the risk of anxiety outcomes were performed with the category that was considered to possess the most anti-inflammatory potential as the referent. Joint effects of consuming the pro-inflammatory diet in combination with each pro-inflammatory lifestyle on incident anxiety outcomes (total anxiety and primary types) were estimated by testing both additive and multiplicative interactions in the multivariable-adjusted Cox models [44]. The HRs and 95% CIs were computed in each joint group of binary E-DII groups (the median value as the cutoff) and an inflammation-related lifestyle when treating subjects at the lower E-DII level and the most anti-inflammatory lifestyle as the referent. The additive interactions were measured by relative excess risk due to interaction (RERI), considered to be the best choice of measures of additivity using a proportional hazards model, under the null hypothesis that RERI = 0 [44]. For multiplicative interactions, we calculated the expected HR as the product of the independent effects from the higher E-DII group and each inflammation-related lifestyle, and compared it with the observed HR from the joint group of both risk factors, under the null hypothesis that the observed HR is less than or equal to the expected HR. The *p*-value for multiplicative interaction was calculated by adding the cross-product of binary E-DII and each lifestyle in the multivariable Cox model, and *p* < 0.10 was considered statistically significant for the interaction analyses.

Several sensitivity analyses were performed. First, we applied E-DII scores computed by using 28 food parameters derived directly from the UK Biobank. Second, to improve the representativeness of participants’ dietary data, we only included participants with more than two rounds of 24 h dietary recalls on at least one weekday and one weekend day, and whose diets were typical and did not have large week-to-week variation (response of “often” to question “Does your diet vary much from week to week?” (Field 1548)). Third, to minimize the risk of potential reverse causality, we removed those who developed any anxiety disorder in the first two years. Fourth, we excluded participants with missing covariates. Fifth, we excluded participants who had any anxiety-related disorders at baseline, with these disorders displayed in the Supplementary Method section [40]. Sixth, we added reaction to severe stress and adjustment disorders (ICD-10 F43) to the outcome of total anxiety disorder as this disease category comprised post-traumatic stress disorder, another subtype of anxiety. Finally, we excluded participants with anxiety outcomes confirmed by self-report only.

All analyses were performed using SAS software (version 9.4, Cary, NC, USA). All tests were two-sided, with *p*-values < 0.05 considered to be statistically significant unless otherwise noted.

## 3. Results

### 3.1. Baseline Population Characteristics

E-DII scores from diet ranged from −6.27 to 6.06 in this study population, among which 48.59%, 33.35%, 15.83%, and 2.23% of participants completed 2, 3, 4, and 5 rounds of WebQs, respectively. Baseline characteristics of participants according to quartiles of E-DII are presented in Table 1. Compared with participants consuming the most anti-inflammatory diet (i.e., E-DII quartile 1), those consuming diets with more pro-inflammatory potential were younger, less physically active, less depressed, more socially deprived, and were more likely to be male, Asian or Asian British, or of mixed ethnicities, current drinkers and smokers, NSAIDs users, non-supplement users, and more likely to have higher BMI, greater total energy intake, lower educational attainment, poorer sleep quality, and more comorbidities.

### 3.2. Associations between E-DII Scores and Incident Anxiety Outcomes

After a median of 9.4 years of follow-up, a total of 2785 incident cases of anxiety disorders occurred (311 cases of phobic anxiety disorders, 16 OCD cases, and 2526 cases of other anxiety disorders, which included three subtypes: 171 cases of panic disorder, 301 cases of mixed anxiety and depressive disorder, and 1824 cases of unspecified anxiety disorder). HRs and 95% CIs for anxiety outcomes across E-DII quartiles as well as a continuous measure with a one-unit increase of SD of E-DII are presented in Table 2. After adjusting for all the covariates, subjects consuming a more proinflammatory diet had a significantly higher total anxiety risk (HR_Q4vsQ1_ = 1.12, 95% CI = 1.00–1.25, P-trend = 0.04), and this positive association was significant among women only (HR_Q4vsQ1_ = 1.15, 95% CI = 1.00–1.31, *p*-trend = 0.02). Associations based on E-DII as a continuous variable showed a significant 4% (95% CI = 0–8%) and 6% (95% CI = 1–11%) increased risk of total anxiety disorders for each one SD increment of E-DII among all the participants and women only. When examining primary types of anxiety disorders, similar HRs were observed for phobic anxiety disorder (HR_Q4vsQ1_ = 1.19, 95% CI = 0.86–1.65) and other anxiety disorders (HR_Q4vsQ1_ = 1.11, 95% CI = 0.99–1.24), though confidence intervals included the null. In terms of subtypes under other anxiety disorders, a significant positive linear association with E-DII score was observed for panic disorder (HR_one-SD-increment_ = 1.22, 95% CI = 1.04–1.4) and mixed anxiety and depressive disorder (HR_one-SD-increment_ = 1.15, 95% CI = 1.02–1.30). The positive trends for both diseases were significant in women only. E-DII was not related to the largest subtype (i.e., unspecified anxiety disorder). No significant interaction by sex was found for E-DII with any anxiety outcome (Table 2). 

### 3.3. Stratified Associations between E-DII Scores and Incident Anxiety Outcomes

The associations between E-DII scores and incident anxiety outcomes, stratified by other sociodemographic factors and the number of comorbidities, are presented in the Supplementary Tables S1–S3. Statistically significant positive associations of E-DII quartiles with total and other anxiety disorders were identified only among those with a college or university degree, the most deprived, and those with three or more comorbidities at baseline. For phobic anxiety disorders, positive associations with E-DII quartiles were only significant in the group with ≥3 comorbidities. No significant interactions between E-DII quartiles and any of these covariates were identified regarding the risk of any of the three anxiety outcomes.

### 3.4. Joint Effects of Binary E-DII Groups and Inflammation-Related Lifestyle Factors on the Risk of Anxiety Outcomes

The multivariable-adjusted associations between baseline inflammation-related lifestyle factors and the risk of total and primary types of anxiety disorders are presented in Supplementary Table S4. Current smoking (HRs from 1.57 to 1.76) significantly increased the risk across all anxiety outcomes compared to never smoking, while past smoking significantly increased only the risk of total and other anxiety disorders but not phobic anxiety disorders.

**Figure 2:**
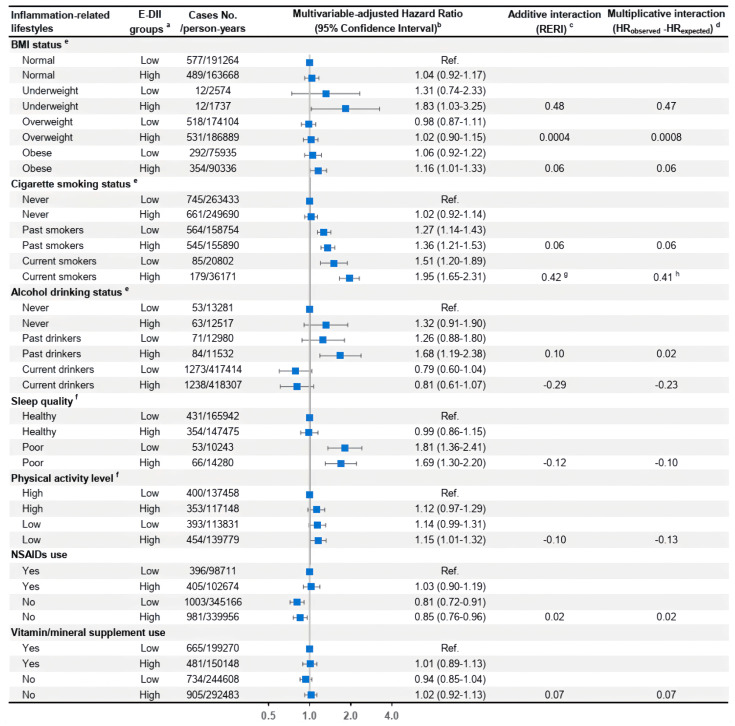
**Joint effects of binary E-DII groups and each inflammation-related lifestyle factor on the risk of total anxiety disorders in the UK Biobank**. Abbreviations: BMI, body mass index; E-DII, energy-adjusted dietary inflammatory index; HR, hazard ratio; MV, multivariable; NSAIDs, nonsteroidal anti-inflammatory drugs; RERI, relative excess risk due to interaction. ^a^ E-DII scores were divided into two groups using the median (−0.03) as the cutoff with the low E-DII group as the reference level. ^b^ The multivariable-adjusted Cox model was adjusted for age group, average total energy, E-DII groups, ethnicity, education qualification, Townsend deprivation index, cigarette smoking status, alcohol, drinking status, physical activity level, BMI status, supplement use, and the number of related comorbidities at baseline, with all these variables defined in the same way as those listed in Table 1. HRs and 95% CIs of incident total anxiety for each combined group of the binary E-DII joined with a baseline inflammation-related lifestyle were calculated when treating subjects at the low E-DII group, with the most anti-inflammatory level of the lifestyle as the reference. ^c^ RERI was calculated to estimate the additive interaction. All *p*-values > 0.10 in this figure if not specified. ^d^ Multiplicative interaction was obtained by the difference between the observed HR for the joint association and the expected HR for the joint effect of two independent risk factors. The *p*-value for multiplicative interaction was calculated with the cross-product of binary E-DII and the inflammation-related lifestyle in the multivariable-adjusted Cox model. All *p*-values > 0.10 in this figure if not specified. ^e^ These inflammation-related lifestyles are all nominal variables with more than two levels, and the additive and multiplicative interaction indicators and corresponding *p*-values for each group compared to the reference lifestyle group were reported as separate independent risk factors. ^f^ For these two lifestyle variables, we only included the two extreme levels as these are ordinal variables. ^g^ *p*-value = 0.06. ^h^ *p*-value = 0.10.

In the joint-effect analyses of binary E-DII groups in conjunction with each inflammation-related lifestyle on total anxiety risk, current smokers who consumed a more pro-inflammatory diet (defined as E-DII above the median value) had a 1.95 times (95% CI = 1.65–2.31) higher risk of total anxiety compared with participants who never smoked and consumed a more anti-inflammatory diet. There was evidence of additive interaction (RERI = 0.42, *p* = 0.06) and multiplicative interaction (HR_observed_–HR_expected_ = 0.41, *p* = 0.10) between a more proinflammatory diet and current smoking status in increasing total anxiety risk. No joint effects of a high E-DII score and other inflammation-related lifestyles were observed on total anxiety risk (Figure 2). Similar additive and multiplicative interactions were present between current smoking status and a higher E-DII on other anxiety disorders (Supplementary Table S5). When investigating the joint effects on total anxiety disorders among men and women separately, similar synergism of current smoking status and a proinflammatory diet under both additive (*p* = 0.05) and multiplicative scales (*p* = 0.09) was observed among women only (Supplementary Figures S1 and S2).

### 3.5. Sensitivity Analyses

The multivariable-adjusted HRs did not change materially in the sensitivity analyses, although the positive association between E-DII and the total anxiety risk was attenuated and not statistically significant after only including participants whose diets did not have large week-to-week variation and who incorporated both weekday and weekend day diets (HR_Q4vsQ1_ =1.13, 95% CI = 0.97–1.33, *p*-trend = 0.28) (Supplementary Figure S3).

## 4. Discussion

In this large prospective cohort study in the UK Biobank, consuming a more pro-inflammatory diet at baseline was significantly associated with an increased incident risk of total anxiety disorders, with positive associations consistently observed with primary types and subtypes of anxiety disorders. The risk effect of E-DII on anxiety disorders was only found in women. Joint effect analyses suggested that current smoking status may synergize with a pro-inflammatory diet to enhance the risk of total anxiety disorders.

Previous studies that investigated dietary inflammatory potential and anxiety outcomes among different populations were all cross-sectional and focused on survey-based anxiety symptoms. Similar to our study, significant positive associations were identified in most studies and were mostly, or most strongly, among women. Among 7083 Iranian adults, severe anxiety symptoms assessed from the Beck Anxiety Inventory were associated with the most proinflammatory diet (OR_Q4 vs Q1_ = 1.33, 95% CI = 1.00–1.78), with the statistically significant association only found in women [14]. In another Iranian study of 3363 adults with a mean age of 36.2 years where anxiety symptoms were evaluated by the Hospital Anxiety and Depression Scale (HADS), the highest versus the lowest DII quintile was related to a significant 1.69-fold odds of anxiety [11]. Based on the same Iranian population, a higher empirically derived food-based dietary inflammatory index score was also found to be significantly associated with a higher level of anxiety in women only [13]. Among older Irish adults at a mean age of 59, the positive association between E-DII tertiles and anxiety symptoms was significant in women as well (OR = 1.60, 95% CI = 1.15–2.24) [10]. Leveraging a 21-item Depression, Anxiety, and Stress Scale, an approximately 3-fold increased anxiety risk was observed among dormitory-residing female university students who were at the highest E-DII tertile [12].

Mental disorders are more prevalent among women than men [2]. Sex differences in the association between the inflammatory potential of the diet and anxiety may arise from a combination of hormonal, genetic, and psychosocial factors [13]. Epidemiological and experimental immunological evidence suggests estrogen enhances humoral immunity; thus, immune markers and inflammatory markers tend to be higher in females [45]. Plus, consumption of a pro-inflammatory diet increases the level of inflammatory markers, which may lead to a stronger DII-related anxiety in females. There were also sex differences in personality traits and psychosocial factors. Women are more likely to be anxious about their body size and health behaviors; therefore, women consuming more pro-inflammatory diets who are more likely to be obese might have a higher anxiety level than men [2]. However, biological processes/mechanisms should be explored in-depth to clarify the sex disparity underlying the diet and mental health relationship.

When examining three subtypes under the primary type of other anxiety disorders, panic disorder and mixed anxiety and depressive disorder had significant positive trends across E-DII quartiles with a larger magnitude of estimate (adjusted HRs = 1.48 and 1.52 respectively) compared to total anxiety disorders and the other subtype (i.e., unspecific anxiety disorder). Therefore, it was presumed that these two subtypes may drive the observed significant positive associations of E-DII with total and other anxiety disorders. However, because patients with anxiety disorders are mostly seen in primary care, the small case number for these two subtypes resulting from only including in-hospital patients warrants future investigations. In addition, these two primary types of anxiety disorders are etiologically heterogeneous, given prevalence rates of both primary types were highest in early or middle adulthood and were lowest in the elderly (65 to 79 years) [46], so future cohort studies with a younger age range are needed to investigate and replicate the dietary associations with various specific types of anxiety disorders.

There are multiple mechanisms by which pro-inflammatory diets may affect anxiety disorders. MGBA, a bidirectional communication system between the gut and the central nervous system, is presumably a fundamental link between diet and mental disorders. Depression-associated dietary patterns are in line with changes in microbial composition and functions, which MGBA research shows can affect emotional behavior in rodents [7,47]. An anti-inflammatory diet with higher dietary fiber intake could result in increased production of short-chain fatty acids (SCFAs) in the gut, especially butyrate, which contribute to reduced systemic inflammation and neuroinflammation by increasing gut epithelial integrity and reducing the translocation of pro-inflammatory stimuli into systemic circulation [7]. SCFAs stimulate the secretion of serotonin in the gut, which can activate the vagus nerve, and enter the circulation to modulate anxiety symptoms [7], and they also activate free fatty acid receptors (FFARs), which have a direct anti-inflammatory effect on microglial activation [48]. In addition, an anti-inflammatory diet provides nutrients (e.g., B vitamins, vitamin C, vitamin E, magnesium, and zinc) that can affect anxiety risk through effects on the production and metabolism of neurotransmitters such as serotonin and noradrenaline, alterations to the hypothalamic-pituitary-adrenal (HPA) system, neuronal membrane structure, or oxidative and nitrosative stress (ONS), all of which have been implicated in psychiatric disorders [4,7,49,50,51]. Dietary habits have been shown to drastically affect the number and composition of circulating monocytes, monocyte migration, and cytokine production, and activated monocytes/macrophages traffic primarily to perivascular and meningeal spaces during peripheral inflammation, which has been shown to contribute to behavioral changes in rodent models of stress-induced depressive and anxiety behaviors [49].

This study suggested a positive current smoking status may synergize with a pro-inflammatory diet to enhance total anxiety. This joint effect was even stronger in women. Cigarette smoking has been shown to augment the production of pro-inflammatory cytokines such as IL-1, IL-6, and IL-8 and to decrease the levels of anti-inflammatory cytokines such as IL-10 [52]. One of the key mechanisms behind smoking-induced inflammation activation is through the NF-κB pathway [53], which serves as the common mechanism through which beneficial dietary components reduce inflammation. Although the reasons for sexual difference in the smoking and DII interaction were not clear, substantial evidence suggests that the smoking behaviors of women, relative to that of men, is less sensitive to manipulations of nicotine and more sensitive to non-nicotine factors, such as smoking-associated environmental stimuli (e.g., stressors) [54]. Therefore, the observed synergistic effect with a pro-inflammatory diet for female smokers could be from some underling cues of smoking (e.g., chronic stress or other interactive lifestyles).

Strengths of the study included a large and well-characterized prospective cohort with a long follow-up to establish temporality in the relationship between dietary intake and incident anxiety outcomes as well as enable sex-specific analyses. The detailed collection of important confounders for adjustment and the utilization of repeated measures of 24 h dietary recalls to well represent an individual’s diet habits were other strengths. Compared to anxiety symptoms assessed in most previous studies, the use of clinically diagnosed anxiety outcomes based on ICD codes allowed for comparable and accurate definitions and assessments of anxiety disorders, and facilitated analyses related to different types of anxiety disorders. Careful sensitivity analyses were conducted to address potential biases, producing robust results to support the main findings. Several limitations should be noted. Firstly, the small case number of OCD and other subtypes such as GAD as a common anxiety disorder precluded analyses for their associations with E-DII. Secondly, it should be noted that anxiety disorders are highly comorbid with other anxiety disorders and other mental disorders, so we cannot fully disentangle incident anxiety risk from other comorbid mental illnesses when interpretating the results. Thirteen food parameters were not available in the E-DII calculation, and all of these components were anti-inflammatory, which could have led to a non-differential misclassification. However, as previously noted, the range of DII scores may rely more on the amount of intake of components rather than on the number of DII components included [55]. Residual or unmeasured confounding was another important limitation, as variables such as perceived social support and significant life events may both influence diet and anxiety but were not collected. In addition, the generalizability of findings should be made with caution, as this study population had limited racial and ethnic diversity to allow for considerations of cultural context. Last but not least, the participants in this study were older, with a median age of 58 years; however, anxiety prevalence is highest in early or middle adulthood, so large prospective cohort studies with younger adults may be needed to replicate these study findings.

## 5. Conclusions

The inflammatory potential of the diet refers to the ability of the diet to influence inflammatory processes in the body. In this prospective cohort study, consuming a diet with more pro-inflammatory potential at baseline was associated with an increased incident risk of anxiety disorders, including total anxiety disorders, panic disorders, and mixed anxiety and depression disorders. These positive associations were predominantly seen in women. Current smoking status may synergize with a pro-inflammatory diet to enhance the risk of total anxiety disorders. This synergistic effect was only observed in females. Our findings suggest that certain dietary patterns may have pro-inflammatory or anti-inflammatory effects, which could be linked to mental disorders such as anxiety. For the general population, and particularly for women, consuming an anti-inflammatory diet may be beneficial in preventing anxiety disorders, and smoking cessation is encouraged for those who consume a pro-inflammatory diet in order to reduce their chances of developing anxiety. Future large prospective cohort studies among diverse populations, especially those with younger ages, collecting detailed clinical information on specific types and subtypes of anxiety disorders, are warranted to confirm our study findings. Biological mechanisms through which diet impacts anxiety development, and diet interacts with other lifestyles to enhance anxiety risk via chronic inflammation, should be explored in depth.

## Figures and Tables

**Table 1:** Baseline characteristics of 96,679 participants by quartiles of E-DII scores in the UK Biobank.

Characteristics ^1^	E-DII Scores from Diet ^2^				
	Q1	Q2	Q3	Q4	*p*-Value ^3^
**E-DII score range (min, max)**	–6.27, –1.40	–1.39, –0.03	–0.02, 1.29	1.30, 6.06	
**N**	24,170	24,170	24,170	24,169	
	**Mean (SD)**				
**Age, years**	57.28 (7.45)	56.96 (7.66)	56.27 (7.90)	55.37 (8.07)	<0.001
**Average total energy intake, kcal/day ^4^**	1865.85 (440.72)	1992.27 (452.66)	2091.82 (477.10)	2225.64 (533.89)	<0.001
**BMI, kg/m^2^**	26.27 (4.52)	26.39 (4.38)	26.67 (4.39)	27.15 (4.64)	<0.001
	**N (%) ^5^**				
**Sex**					<0.001
Male	7503 (31.04)	9911 (41.01)	11,981 (49.57)	14,289 (59.12)	
Female	16,667 (68.96)	14,259 (58.99)	12,189 (50.43)	9880 (40.88)	
**Ethnicity**					<0.001
Asian or Asian British	208 (0.86)	187 (0.77)	229 (0.95)	255 (1.06)	
Black or Black British	205 (0.85)	136 (0.56)	165 (0.68)	210 (0.87)	
Chinese	70 (0.29)	66 (0.27)	63 (0.26)	51 (0.21)	
Mixed	131 (0.54)	92 (0.38)	119 (0.49)	145 (0.60)	
Other ethnic groups	169 (0.70)	114 (0.47)	126 (0.52)	129 (0.53)	
White	23,321 (96.49)	23,500 (97.23)	23,397 (96.80)	23,279 (96.32)	
Unknown	66 (0.27)	75 (0.31)	71 (0.29)	100 (0.41)	
**Education qualification ^6^**					<0.001
College or university degree/vocational qualification	17,729 (73.35)	17,817 (73.72)	17,698 (73.22)	16,995 (70.32)	
National examination at age 17–18	1453 (6.01)	1477 (6.11)	1505 (6.23)	1564 (6.47)	
National examination at age 16	3303 (13.67)	3203 (13.25)	3265 (13.51)	3685 (15.25)	
Unknown	1685 (6.97)	1673 (6.92)	1702 (7.04)	1925 (7.96)	
**Townsend deprivation index ^7^**					<0.001
Least deprived, −6.26–−3.32	8149 (33.72)	8352 (34.56)	8158 (33.75)	7535 (31.18)	
Intermediate, −3.31–−1.09	8093 (33.48)	8259 (34.17)	8027 (33.21)	7815 (32.33)	
Most deprived, –1.08–10.27	7908 (32.72)	7534 (31.17)	7956 (32.92)	8794 (36.39)	
Unknown	20 (0.08)	25 (0.10)	29 (0.12)	25 (0.10)	
**Cigarette smoking status**					<0.001
Never	14,247 (58.94)	14,347 (59.36)	14,011 (57.97)	13,039 (53.95)	
Past smokers, ≥15 cigarettes/d	3725 (15.41)	3651 (15.11)	3888 (16.09)	4130 (17.09)	
Past smokers, <15 cigarettes/d	1748 (7.23)	1649 (6.82)	1586 (6.56)	1465 (6.06)	
Past smokers, amount unknown	3319 (13.73)	3260 (13.49)	3145 (13.01)	2921 (12.09)	
Current smokers, ≥15 cigarettes/d	222 (0.92)	292 (1.21)	418 (1.73)	1010 (4.18)	
Current smokers, <15 cigarettes/d	343 (1.42)	364 (1.51)	424 (1.75)	712 (2.95)	
Current smokers, amount unknown	512 (2.12)	565 (2.34)	658 (2.72)	838 (3.47)	
Unknown status	54 (0.22)	42 (0.17)	40 (0.17)	54 (0.22)	
**Alcohol drinking status ^7^**					<0.001
Never	818 (3.38)	646 (2.67)	645 (2.67)	738 (3.05)	
Past drinkers	802 (3.32)	636 (2.63)	561 (2.32)	743 (3.07)	
Current drinkers, ≤7.1 g/d	8206 (33.95)	7557 (31.27)	7157 (29.61)	7423 (30.71)	
Current drinkers, 7.2–18.6 g/d	8025 (33.20)	8042 (33.27)	7614 (31.50)	6665 (27.58)	
Current drinkers, >18.6 g/d	6306 (26.09)	7280 (30.12)	8184 (33.86)	8580 (35.50)	
Unknown status	13 (0.05)	9 (0.04)	9 (0.04)	20 (0.08)	
**Sleep quality ^8^**					
Healthy	9174 (37.96)	8835 (36.55)	8381 (34.68)	7638 (31.60)	<0.001
Intermediate	10,701 (44.27)	10,891 (45.06)	11,167 (46.20)	11,619 (48.07)	
Poor	525 (2.17)	607 (2.51)	684 (2.83)	900 (3.72)	
Unknown	3770 (15.60)	3837 (15.88)	3938 (16.29)	4012 (16.60)	
**Physical activity level ^7,9^**					<0.001
Low, ≤1102 MET-minutes/week	5799 (23.99)	6643 (27.48)	7274 (30.10)	8022 (33.19)	
Medium, 1103–2604 MET-minutes/week	7115 (29.44)	7161 (29.63)	6977 (28.87)	6457 (26.72)	
High, >2604 MET-minutes/week	7916 (32.75)	7013 (29.02)	6496 (26.88)	6267 (25.93)	
Unknown	3340 (13.82)	3353 (13.87)	3423 (14.16)	3423 (14.16)	
**BMI status**					<0.001
Underweight, <18.5 kg/m^2^	165 (0.68)	116 (0.48)	89 (0.37)	105 (0.43)	
Normal weight, 18.5–24.9 kg/m^2^	10,600 (43.86)	10,165 (42.06)	9391 (38.85)	8416 (34.82)	
Overweight, 25.0–29.9 kg/m^2^	9233 (38.20)	9695 (40.11)	10,123 (41.88)	10,258 (42.44)	
Obese, ≥30 kg/m^2^	4172 (17.26)	4194 (17.35)	4567 (18.9)	5390 (22.30)	
**Vitamin/mineral supplement use**					<0.001
No	12,700 (52.54)	13,908 (57.54)	15,178 (62.80)	16,706 (69.12)	
Yes	11,470 (47.46)	10,262 (42.46)	8992 (37.20)	7463 (30.88)	
**NSAIDs use**					<0.001
No	18,799 (77.78)	18,706 (77.39)	18,590 (76.91)	18,395 (76.11)	
Yes	5371 (22.22)	5464 (22.61)	5580 (23.09)	5774 (23.89)	
**Depression status**					<0.001
No	20,997 (86.87)	21,339 (88.29)	21,303 (88.14)	21,141 (87.47)	
Yes	3173 (13.13)	2831 (11.71)	2867 (11.86)	3028 (12.53)	
**Number of related comorbidities ^10^**					0.03
0	10,631 (43.98)	10,682 (44.20)	10,759 (44.51)	10,683 (44.20)	
1–2	11,048 (45.71)	10,913 (45.15)	10,847 (44.88)	10,778 (44.59)	
≥3	2491 (10.31)	2575 (10.65)	2564 (10.61)	2708 (11.2)	

Abbreviations: ANOVA, Analysis of Variance; BMI, body mass index; E-DII, energy-adjusted dietary inflammatory index; GCSE, General Certificate of Secondary Education; IPAQ, International Physical Activity Questionnaire; MET: Metabolic Equivalent Task; NSAIDs, nonsteroidal anti-inflammatory drugs; SD, standard deviation. ^1^ The detailed assessment and categorization of baseline characteristics listed in this table are described in the Supplementary Method section. ^2^ The E-DII score from the diet of each individual was derived by linking DII with the average energy-adjusted dietary intake of 32 food parameters included in the DII from all rounds of 24 h dietary recalls with the typical diet indicated. ^3^ The *p*-value was calculated by ANOVA test for continuous variables and Chi-square test for categorical variables. ^4^ The average total energy intake for each individual was calculated by averaging the total energy intake from all the included rounds of 24 h dietary recalls. ^5^ Percentages for each E-DII quartile may not add up to 100% due to rounding. ^6^ ‘National examination at age 17–18’ refers to the intermediate qualifications, including ‘A levels/AS levels or equivalent’, and ‘National examination at age 16’ refers to the lowest qualifications, including ‘O levels/GCSEs or equivalent’ and ‘CSEs or equivalent’. ^7^ These characteristics were categorized into tertiles based on the population distribution at baseline. We only categorized daily alcohol intake among current drinkers because of data availability in this group only. ^8^ Sleep quality was assessed by a healthy sleep score, which was calculated by summing the number of low-risk sleep factors self-reported on the touchscreen questionnaire: (1) sleeping 7–8 h per day, (2) early chronotype (‘definitely a morning person’ or ‘more a morning than evening person’), (3) reported insomnia symptoms (never or rarely), (4) not reporting snoring, and (5) not reporting frequent daytime sleepiness (‘never/rarely’ or ‘sometimes’). For each factor, participants received a score of 1 if they had low risk for that factor. Sleep quality was defined as ‘poor’ (healthy sleep score ≤ 1), ‘intermediate’ (2 ≤ healthy sleep score ≤ 3), ‘healthy’ (healthy sleep score ≥ 4), and ‘unknown’ (any factor was missing). ^9^ Total physical activity was derived by summing MET-minutes per week for all activities, including walking, moderate, vigorous activity, and categorized into tertiles. ^10^ A total of seven chronic diseases at baseline related to diet and anxiety were included: cardiovascular diseases, hypertension, hyperlipidemia, type-2 diabetes mellitus, cancers, digestive diseases, and chronic kidney diseases. The number of related comorbidities was the sum of the seven diseases an individual was diagnosed at baseline based on self-reported medical conditions, medication use, and hospital inpatient records.

**Table 2:** Associations between E-DII scores from diet and incident anxiety outcomes in the UK Biobank.

	Quartile 1	Quartile 2	Quartile 3	Quartile 4	*P_trend_* ^1^	HR_continuous_ (95% CI) ^2^	*P_interaction-by-sex_* ^3^
**Total anxiety disorders ^4^**							0.57
No. of cases/person-years	722/222,038	677/221,840	647/221,779	739/220,852			
Model 1, HR (95% CI) ^5^	Ref.	0.99 (0.89–1.10)	1.00 (0.90–1.11)	1.21 (1.09–1.35)	<0.001	1.08 (1.04–1.12)	
Model 2, HR (95% CI) ^6^	Ref.	1.00 (0.90–1.11)	0.99 (0.89–1.10)	1.12 (1.00–1.25)	0.04	1.04 (1.00–1.08)	
**Males**							
No. of cases/person-years	145/68,717	202/90,872	218/109,875	320/130,288			
Model 1, HR (95% CI) ^5^	Ref.	1.05 (0.85–1.31)	0.94 (0.76–1.16)	1.17 (0.95–1.43)	0.23	1.04 (0.97–1.12)	
Model 2, HR (95% CI) ^6^	Ref.	1.05 (0.85–1.31)	0.94 (0.76–1.17)	1.08 (0.88–1.32)	0.95	1.00 (0.94–1.07)	
**Females**							
No. of cases/person-years	577/153,321	475/130,968	429/111,904	419/90,564			
Model 1, HR (95% CI) ^5^	Ref.	0.96 (0.85–1.09)	1.02 (0.90–1.16)	1.24 (1.09–1.41)	<0.001	1.09 (1.04–1.14)	
Model 2, HR (95% CI) ^6^	Ref.	0.97 (0.86–1.10)	1.01 (0.89–1.15)	1.15 (1.00–1.31)	0.02	1.06 (1.01–1.11)	
**Phobic anxiety disorders ^4^**							0.84
No. of cases/person-years	80/224,777	84/224,242	63/223,988	84/223,457			
Model 1, HR (95% CI) ^5^	Ref.	1.13 (0.83–1.53)	0.91 (0.65–1.27)	1.32 (0.96–1.82)	0.03	1.14 (1.01–1.28)	
Model 2, HR (95% CI) ^6^	Ref.	1.13 (0.83–1.53)	0.88 (0.63–1.24)	1.19 (0.86–1.65)	0.17	1.09 (0.97–1.22)	
**Males**							
No. of cases/person-years	17/69,214	20/91,577	20/110,589	37/131,432			
Model 1, HR (95% CI) ^5^	Ref.	0.90 (0.47–1.72)	0.76 (0.40–1.46)	1.21 (0.67–2.18)	0.18	1.15 (0.93–1.43)	
Model 2, HR (95% CI) ^6^	Ref.	0.90 (0.47–1.71)	0.75 (0.39–1.44)	1.12 (0.61–2.05)	0.31	1.12 (0.90–1.39)	
**Females**							
No. of cases/person-years	63/155,563	64/132,664	43/113,399	47/92,024			
Model 1, HR (95% CI) ^5^	Ref.	1.20 (0.85–1.70)	0.96 (0.65–1.41)	1.32 (0.90–1.95)	0.10	1.12 (0.98–1.29)	
Model 2, HR (95% CI) ^6^	Ref.	1.21 (0.85–1.71)	0.93 (0.63–1.38)	1.19 (0.80–1.76)	0.30	1.08 (0.94–1.24)	
**Other anxiety disorders ^4^**							0.39
No. of cases/person-years	656/222,306	605/222,174	597/221,994	668/221,192			
Model 1, HR (95% CI) ^5^	Ref.	0.97 (0.87–1.09)	1.01 (0.90–1.13)	1.20 (1.07–1.34)	<0.001	1.07 (1.03–1.12)	
Model 2, HR (95% CI) ^6^	Ref.	0.98 (0.87–1.09)	1.00 (0.89–1.12)	1.11 (0.99–1.24)	0.09	1.04 (0.995–1.08)	
**Males**							
No. of cases/person-years	130/68,775	185/90,943	203/109,948	286/130,453			
Model 1, HR (95% CI) ^5^	Ref.	1.08 (0.86–1.35)	0.98 (0.78–1.22)	1.16 (0.94–1.43)	0.43	1.03 (0.96–1.11)	
Model 2, HR (95% CI) ^6^	Ref.	1.08 (0.86–1.35)	0.98 (0.78–1.22)	1.07 (0.86–1.33)	0.78	0.99 (0.92–1.06)	
**Females**							
No. of cases/person-years	526/153,532	420/131,231	394/112,047	382/90,739			
Model 1, HR (95% CI) ^5^	Ref.	0.93 (0.82–1.06)	1.03 (0.90–1.17)	1.23 (1.08–1.41)	<0.001	1.09 (1.04–1.15)	
Model 2, HR (95% CI) ^6^	Ref.	0.94 (0.82–1.07)	1.01 (0.89–1.15)	1.14 (0.99–1.30)	0.03	1.06 (1.01–1.11)	
**Panic disorder^7^**							0.99
No. of cases/person-years	38/224,913	35/224,472	44/224,095	54/223,694			
Model 1, HR (95% CI) ^5^	Ref.	0.97 (0.61–1.53)	1.27 (0.82–1.97)	1.66 (1.09–2.54)	0.002	1.28 (1.10–1.50)	
Model 2, HR (95% CI) ^6^	Ref.	0.98 (0.62–1.55)	1.26 (0.81–1.97)	1.48 (0.95–2.31)	0.02	1.22 (1.04–1.43)	
**Males**							
No. of cases/person-years	10/69,243	12/91,605	18/110,630	27/131,547			
Model 1, HR (95% CI) ^5^	Ref.	0.92 (0.40–2.13)	1.18 (0.55–2.56)	1.57 (0.76–3.24)	0.07	1.26 (0.98–1.61)	
Model 2, HR (95% CI) ^6^	Ref.	0.92 (0.40–2.14)	1.19 (0.54–2.61)	1.40 (0.66–2.97)	0.19	1.19 (0.92–1.53)	
**Females**							
No. of cases/person-years	28/155,669	23/132,867	26/113,465	27/92,147			
Model 1, HR (95% CI) ^5^	Ref.	0.97 (0.56–1.69)	1.30 (0.76–2.23)	1.70 (1.00–2.89)	0.01	1.29 (1.06–1.57)	
Model 2, HR (95% CI) ^6^	Ref.	0.98 (0.56–1.70)	1.28 (0.74–2.20)	1.53 (0.88–2.67)	0.04	1.23 (1.01–1.51)	
**Mixed anxiety and depressive disorder ^7^**							0.81
No. of cases/person-years	65/224,809	67/224,381	73/223,950	96/223,478			
Model 1, HR (95% CI) ^5^	Ref.	1.08 (0.77–1.53)	1.23 (0.88–1.73)	1.71 (1.24–2.36)	0.001	1.22 (1.08–1.37)	
Model 2, HR (95% CI) ^6^	Ref.	1.08 (0.76–1.52)	1.20 (0.85–1.68)	1.52 (1.09–2.13)	0.02	1.15 (1.02–1.30)	
**Males**							
No. of cases/person-years	17/69,210	20/91,595	28/110,570	43/131,456			
Model 1, HR (95% CI) ^5^	Ref.	0.89 (0.46–1.69)	1.02 (0.56–1.86)	1.29 (0.74–2.27)	0.23	1.13 (0.93–1.36)	
Model 2, HR (95% CI) ^6^	Ref.	0.87 (0.45–1.66)	0.97 (0.53–1.78)	1.08 (0.60–1.94)	0.74	1.03 (0.85–1.26)	
**Females**							
No. of cases/person-years	48/155,598	47/132,786	45/113,380	53/92,023			
Model 1, HR (95% CI) ^5^	Ref.	1.16 (0.78–1.74)	1.32 (0.88–1.98)	1.95 (1.31–2.88)	0.001	1.26 (1.09–1.45)	
Model 2, HR (95% CI) ^6^	Ref.	1.17 (0.78–1.76)	1.31 (0.87–1.98)	1.81 (1.20–2.73)	0.01	1.22 (1.05–1.41)	
**Unspecified anxiety disorder ^7^**							0.31
No. of cases/person-years	486/223,364	429/223,209	439/222,867	470/222,286			
Model 1, HR (95% CI) ^5^	Ref.	0.95 (0.83–1.08)	1.04 (0.91–1.18)	1.21 (1.06–1.38)	0.002	1.08 (1.03–1.13)	
Model 2, HR (95% CI) ^6^	Ref.	0.94 (0.83–1.07)	1.01 (0.88–1.15)	1.08 (0.94–1.24)	0.26	1.03 (0.98–1.08)	
**Males**							
No. of cases/person-years	93/68,983	129/91,262	142/110,253	191/130,938			
Model 1, HR (95% CI) ^5^	Ref.	1.06 (0.81–1.38)	0.97 (0.75–1.26)	1.12 (0.87–1.43)	0.76	1.01 (0.93–1.10)	
Model 2, HR (95% CI) ^6^	Ref.	1.05 (0.80–1.37)	0.97 (0.74–1.26)	1.02 (0.79–1.32)	0.46	0.97 (0.89–1.06)	
**Females**							
No. of cases/person-years	393/154,380	300/131,947	297/112,614	279/91,348			
Model 1, HR (95% CI) ^5^	Ref.	0.91 (0.78–1.06)	1.07 (0.92–1.24)	1.27 (1.09–1.48)	<0.001	1.11 (1.05–1.17)	
Model 2, HR (95% CI) ^6^	Ref.	0.90 (0.77–1.05)	1.03 (0.88–1.20)	1.12 (0.95–1.32)	0.07	1.05 (0.995–1.12)	

Abbreviations: E-DII, energy-adjusted dietary inflammatory index; HR, hazard ratio; ICD, International Classification of Diseases; OCD, obsessive-compulsive disorder; ^1^ *P_trend_* was calculated using the continuous E-DII score in the corresponding model. ^2^ The continuous HR and associated 95% CI was calculated for each one standard deviation increase in the E-DII score. ^3^ *P_interaction-by-sex_* was calculated with the cross-product of sex and E-DII quartiles in the multivariable-adjusted Cox model (Model 2). ^4^ Total anxiety disorders include phobic anxiety disorders (ICD-10: F40, including agoraphobia, social phobias, specific phobias, other phobic anxiety disorders, unspecified phobic anxiety disorder), other anxiety disorders (ICD-10: F41, including panic disorder, generalized anxiety disorder, mixed anxiety and depressive disorder, other mixed anxiety disorders, other specified anxiety disorders, unspecified anxiety disorder), and OCD (ICD-10: F42, including predominantly obsessional thoughts or ruminations, predominantly compulsive acts, mixed obsessional thoughts and acts, other obsessive-compulsive disorders, unspecified obsessive-compulsive disorder). As the number of OCD cases was only 16, we did not list and analyze. The total number of cases was not the same as the sum of three diseases (F40–F42) because one may have multiple anxiety disorders. ^5^ Model 1 adjusted for age (<58, ≥58), sex (male, female), and average total energy intake (kcal/day). ^6^ Model 2 adjusted for age (<58, ≥58), sex (male, female), average total energy (kcal/day), ethnicity (Asian or Asian British, Black or Black British, Chinese, mixed, other ethnic groups, White, unknown), education qualification (college or university degree/vocational qualification, national examination at age 17–18, national examination at age 16, unknown), Townsend deprivation index (least deprived, intermediate, most deprived, unknown), cigarette smoking status (never, past smokers ≥ 15 cigarettes/d, past smokers < 15 cigarettes/d, past smokers with amount unknown, current smokers ≥ 15 cigarettes/d, current smokers < 15 cigarettes/d, current smokers with amount unknown, unknown status), alcohol drinking status (never, past drinkers, current drinkers ≤ 7.1 g/d, current drinkers with 7.2–18.6 g/d, current drinkers > 18.6 g/d, unknown status), physical activity (low, medium, high, unknown), depression status (yes, no), sleep quality (healthy, intermediate, poor, unknown), body mass index status (underweight, normal, overweight, obese), vitamin/mineral supplement use (no, yes), number of related comorbidities (0, 1–2, ≥3). ^7^ Panic disorder (ICD-10: F41.0), mixed anxiety and depressive disorder (ICD-10: F41.2), and unspecified anxiety disorder (ICD-10: F41.9) are three subtypes of other anxiety disorders. We did not analyze the remaining subtypes of other anxiety disorders due to the small number of cases (35 cases for generalized anxiety disorder, 3 cases for other mixed anxiety disorders, 4 cases for other specified anxiety disorders).

## Data Availability

The datasets used for analysis are under UKB proposal application number 83432. Data availability should be requested from the corresponding author.

## Supplementary Materials

The following supporting information can be downloaded at: https://www.mdpi.com/article/10.3390/nu16010121/s1, Supplementary Information S1: Anxiolytics code classification tables; Supplementary Information S2: Covariates assessment and categorization; Supplementary Information S3: Nonsteroidal anti-inflammatory drugs (NSAIDs) and corresponding drug IDs in the UK Biobank; Supplementary Information S4: The code list of antidepressants in the UK Biobank; Supplementary Information S5: The list of excluded anxiety-related disorders at baseline in the sensitivity analysis; Supplementary Information S6: Calculation of pure alcohol intake in the UK Biobank; Supplementary Information S7: Codes used in the UK Biobank to define prevalent comorbidities at baseline; Supplementary Table S1: Stratified associations between E-DII scores and the risk of total anxiety disorders in the UK Biobank by sociodemographic and comorbidity factors (n = 96,679); Supplementary Table S2: Stratified associations between E-DII scores and the risk of phobic anxiety disorders in the UK Biobank by sociodemographic and comorbidity factors (n = 96,679); Supplementary Table S3: Stratified associations between E-DII scores and the risk of other anxiety disorders in the UK Biobank by sociodemographic and comorbidity factors (n = 96,679); Supplementary Table S4: Multivariable-adjusted associations between baseline inflammation-related lifestyle factors and the risk of anxiety outcomes in the UK Biobank (n = 96,679); Supplementary Table S5: Joint effects of binary E-DII groups and inflammation-related lifestyle factors on the risk of phobic and other anxiety disorders in the UK Biobank (n = 96,679); Supplementary Figure S1: Joint effects of binary E-DII groups and inflammation-related lifestyles on the risk of total anxiety disorders among males in the UK Biobank (n = 43,684); Supplementary Figure S2: Joint effects of binary E-DII groups and inflammation-related lifestyles on the risk of total anxiety disorders among females in the UK Biobank (n = 52,995); Supplementary Figure S3: Sensitivity analyses of multivariable-adjusted associations between E-DII scores and the risk of anxiety outcomes. References [23,33,34,35,36,40,56,57,58,59,60,61,62,63,64,65,66,67,68,69,70,71,72,73,74,75,76,77,78,79] are cited in the supplementary materials.
