# Supplementary material for: Dietary Inflammatory Potential, Inflammation-Related Lifestyle Factors, and Incident Anxiety Disorders: A Prospective Cohort Study
Source: Nutrients. 2023 Dec 29;16(1):121. doi: 10.3390/nu16010121 (PMC10781140; doi:10.3390/nu16010121)
Supplement: Supplementary file 1 [file nutrients-16-00121-s001.zip › nutrients-2767639-supplementary.pdf]

Contents

Supplemental method **2**
Supplementary Information S1. Anxiolytics code classification tables 2
Supplementary Information S2. Covariates assessment and categorization 3
Supplementary Information S3. Nonsteroidal anti-inflammatory drugs (NSAIDs) and corresponding drug IDs in the UK Biobank 6
Supplementary Information S4. The code list of antidepressants in the UK Biobank 6
Supplementary Information S5. The list of excluded anxiety-related disorders at baseline in the sensitivity analysis 8
Supplementary Information S6. Calculation of pure alcohol intake in the UK Biobank 9
Supplementary Information S7. Codes used in the UK Biobank to define prevalent comorbidities at baseline 9
Supplemental Table S1. Stratified associations between E-DII scores and risk of total anxiety disorders in the UK Biobank by sociodemographic and comorbidity factors (n=96679) **11**
Supplemental Table S2. Stratified associations between E-DII scores and risk of phobic anxiety disorders in the UK Biobank by sociodemographic and comorbidity factors (n=96679) **12**
Supplemental Table S3. Stratified associations between E-DII scores and risk of other anxiety disorders in the UK Biobank by sociodemographic and comorbidity factors (n=96679) **13**
Supplemental Table S4. Multivariable-adjusted associations between baseline inflammation-related lifestyle factors and risk of anxiety outcomes in the UK Biobank (n=96679) **15**
Supplemental Table S5. Joint effects of binary E-DII groups and inflammation-related lifestyle factors on the risk of phobic and other anxiety disorders in the UK Biobank (n=96679) **16**
Supplemental Figure S1. Joint effects of binary E-DII groups and inflammation-related lifestyles on the risk of total anxiety disorders among males in the UK Biobank (n=43684) **19**
Supplemental Figure S2. Joint effects of binary E-DII groups and inflammation-related lifestyles on the risk of total anxiety disorders among females in the UK Biobank (n=52995) **20**
Supplemental Figure S3. Sensitivity analyses of multivariable-adjusted associations between E-DII scores and risk of anxiety outcomes **21**
References **21**

Supplemental method

Supplementary Information S1. Anxiolytics code classification tables

Anxiolytics [1]

Drug ID	Drug Name
1140863144	zopiclone
1140863152	diazepam
1140863202	temazepam
1140865016	zolpidem
1140863182	nitrazepam
1140863302	lorazepam
1140883656	hydroxyzine
1140928004	zimovane
1140862810	phenergan
1140882082	promethazine
1140879730	buspirone
1140863286	atarax
1140863442	oxazepam
1140863120	loprazolam
1140863328	chlordiazepoxide
1140863176	lormetazepam
1140863292	ucerax
1140864916	stilnoct
1141157496	diazepam
1140863454	buspar
1140863308	alprazolam
1140863350	librium
1140863310	xanax
1140863440	meprate
1140863112	dalmane
1140909798	clomethiazole
1140863378	meprobamate
1140863028	welldorm
1140867938	amitriptyline+chlordiazepoxide
1140863110	flurazepam
1140863036	heminevrin
1140863372	medazepam
1140867136	neulactil
1140882312	sinequan
1140855870	almazine
1140855832	atensine
1140875434	carisoma
1140863410	chloractil

1140863016	chloral
1140855824	dichloralphenazone
1140855890	dormonoct
1140856040	methyprylone
1140863194	mogadon
1140863106	rohypnol
1140867668	tryptizol

Supplementary Information S2. Covariates assessment and categorization

Age

Age was recorded at the reception of the study (Field 21003). We treated age as a continuous variable.

Ethnicity

Ethnicity was categorized into 7 groups: 'Asian or Asian British', 'Black or Black British', 'Chinese', 'Mixed', 'Other ethnic group', 'White', and 'Unknown' based on an amalgam of sequential branching questions asked about ethnicity as part of the touchscreen questionnaire during the initial assessment center visit (Field 21000). In our study, 'Unknown' included options 'Do not know' and 'Prefer not to answer'.

Education qualification

Education qualification was assessed on the baseline touchscreen questionnaire. Participants were asked 'Which of the following qualifications do you have?' (Field 6138). There were 8 options: 'College or university degree', 'A levels/AS levels or equivalent', 'O levels/GCSEs or equivalent', 'CSEs or equivalent', 'NVQ or HND or HNC or equivalent', 'Other professional qualifications e.g.: nursing, teaching', 'None of the above' and 'Prefer not to answer'. We categorized education qualification into four groups: 'College or university degree/vocational qualification' (the highest qualification level including 'College or university degree', 'NVQ or HND or HNC or equivalent' and 'Other professional qualifications e.g.: nursing, teaching'); 'National examination at age 17-18' (the intermediate qualification level including 'A levels/AS levels or equivalent'); 'National examination at age 16' (the lowest qualification level including 'O levels/GCSEs or equivalent' and 'CSEs or equivalent'); and 'unknown'(including 'None of the above', 'prefer not to answer' and missing) [2].

Townsend deprivation index

Townsend deprivation index (Field 189) was calculated immediately prior to participant joining UK Biobank based on the participants' preceding national census output areas in which their postcode is located using four census variables: percentage of non-home ownership, non-car ownership, unemployment and household overcrowding. It is a measure of material deprivation. Negative values indicate relative affluence with low-level deprivation, while positive values indicate areas with high-level deprivation [3]. We categorized participants into tertiles based on this index as "Least deprived", "Intermediate" and "Most deprived", and treated participants without Townsend index as 'Unknown' [2].

Average total energy intake (kcal/day)

Total energy intake (kcal/day) for an individual was estimated from all the foods and beverages consumption during the previous day based on a web-based 24-h dietary questionnaire developed by the Cancer Epidemiology Unit in Oxford for UK Biobank which contained about 200 foods

and drinks [4]. A participant responded to this questionnaire up to 5 occasions from the initial assessment visit to April 2012. Given we only included participants who completed at least 2 questionnaires each indicating a typical diet intake (Field 100020), we averaged the total energy intake of all the rounds of 24-h diets and treated the average total energy intake as a continuous variable.

Cigarette smoking status

Cigarette smoking status was obtained based on the touchscreen questionnaires at baseline. First, we categorized smoking status into 'Never', 'Past smokers', 'Current smokers', and 'Unknown status' based on data from Field 20116 which summarized the current/past/never smoking status of participants. Second, we obtained the current and past smokers' number of cigarettes smoked per day from these two questions respectively: 'About how many cigarettes do you smoke on average each day?' (Field 3456) and 'About how many cigarettes did you smoke on average each day?' (Field 2887). We categorized smoking status into eight groups ('Never', 'Past smokers, ≥15 cigarettes/d', 'Past smokers, <15 cigarettes/d', 'Past smokers, amount unknown', 'Current smokers, ≥15 cigarettes/d', 'Current smokers, <15 cigarettes/d', 'Current smokers, amount unknown', 'Unknown status' based on the median number of number of cigarettes per day for current and past smokers.

Alcohol drinking status

Alcohol drinking status was derived from the touchscreen questionnaires at baseline. First, we categorized alcohol drinking status into 'Never', 'Past drinkers', 'Current drinkers', and 'Unknown' based on the Field 20117. Second, we calculated the alcohol intake of the current drinkers. Participants were asked 'how often do you drink alcohol?' (Field 1558) which included responses of 'daily or almost daily', 'three or four times a week', 'once or twice a week', 'one to three times a month', 'special occasions only', 'never', 'prefer not to answer'. Based on the responded intake frequency, participants were further asked separately about their monthly and weekly intake of red wine (Field 4407 and Field 1568), glasses of white wine/champagne (Field 4418 and Field 1578), pints of beer (Field 4429 and Field 1588), measures of spirits/liqueurs (Field 4440 and Field 1598), glasses of fortified wine (Field 4451 and Field 1608) and glasses of other alcohol (Field 4462 and Field 5364). According to the previous UK Biobank study [5,6], we calculated daily pure alcohol intake by multiplying the estimated grams of alcohol contained in each type of alcoholic drink by the number of alcoholic drinks consumed per month or per week and adding up from all alcoholic drinkers (See Supplemental Information 6). Due to a large proportion of missing data on monthly or weekly intake of alcoholic drinks for each type of drinks, for participants who had unknown grams/day of alcohol but who reported their intake frequency (Field 1558), we assigned them the median value (g/day) of the corresponding category. The intake of alcohol in grams per day was categorized into tertiles among current drinkers.

Sleep quality

We assessed an individual's sleeping quality by calculating a healthy sleep score based on five sleep questions on the baseline touchscreen questionnaire according to previous UK Biobank studies [7,8]: (1) 'About how many hours of sleep do you get in every 24 h? (Please include naps)' (Field 1160), (2) 'Do you consider yourself to be?' with the responses 'definitely a morning person', 'more a morning than evening person', 'more an evening than a morning person', 'definitely an evening person', 'do not know', 'prefer not to answer' (Field 1180), (3) 'Do you have trouble falling asleep at night or do you wake up in the middle of the night?' with the

responses ‘never/rarely’, ‘sometimes’, ‘usually’, ‘prefer not to answer’ (Field 1200), (4) ‘Does your partner or a close relative or friend complain about your snoring?’ with the options ‘yes’, ‘no’, ‘do not know’, ‘prefer not to answer’ (Field 1210), and (5) ‘How likely are you to doze off or fall asleep during the daytime when you don't mean to? (e.g., when working, reading, or driving)’ with the options ‘never/rarely’, ‘sometimes’, ‘often’, ‘prefer not to answer’, ‘all of the time’ (Field 1220). Low-risk sleep factors were defined as follows: (1) sleeping 7–8 h per day, (2) early chronotype (‘definitely a morning person’ or ‘more a morning than evening person’), (3) reported never or rarely had insomnia symptoms, (4) not reporting snoring, and (5) not reporting frequent daytime sleepiness (‘never/rarely’ or ‘sometimes’). For each factor, participants received a score of 1 if they had low risk for that factor. All scores were summed to obtain the healthy sleep score, with higher scores representing a healthier sleep pattern. Sleeping quality of participants was determined based on this score as ‘Poor’ (healthy sleep score ≤1), ‘Intermediate’ (2≤healthy sleep score ≤3), ‘Healthy’ (healthy sleep score ≥4), and ‘unknown’ if any factor score was missing.

Physical activity

Physical activity was assessed using a list of questions on the touchscreen questionnaire at baseline which were adapted from the validated short-form International Physical Activity Questionnaire (IPAQ) that covers the frequency, intensity and duration of walking, moderate and vigorous activity [9,10]. Time spent in vigorous, moderate and walking activity was weighted by the energy expended for these categories to obtain Metabolic Equivalent Task (MET)-minutes/week of physical activity according to IPAQ data processing and analysis guideline [11]. In this study, we used the derived variable of (MET) minutes per week for all activities (Field 22040), and categorized it into tertiles. Participants without physical activity information were defined as ‘Unknown’.

Vitamin/mineral supplement use

Vitamin/mineral supplement use was assessed via the web-based 24-h dietary recall questionnaire. Participants were asked ‘Did you have any vitamin or mineral supplements yesterday? e.g., Vitamin C, multivitamins, fish oil, calcium supplement.’ (Field 104670). Vitamin and/or mineral use in our study was defined as answering “yes” to this question more than half rounds of the included 24-hour dietary recalls for each subject.

Body mass index (BMI)

BMI (Field 21001) was calculated as weight (kg)/height(m)^2 with weight and height measured at the physical measurement stage at baseline by trained staff, and was categorized as ‘Underweight’ (<18.5 kg/m^2), ‘Normal’ (18.5-24.9 kg/m^2), ‘Overweight’ (25.0-29.9 kg/m^2) ‘Obese’ (≥30 kg/m^2) and ‘Unknown’ based on the World Health Organization criteria [12].

Nonsteroidal anti-inflammatory drugs (NSAIDs) use

NSAIDs use at baseline was assessed by trained members at the verbal interview where participants were inquired about their regular prescription medications (Field 20003). Based on a previous study [13] and the availability of prescription medications in the UK Biobank, regular NSAIDs use in our study was categorized as ‘Yes’, ‘No’ and ‘Unknown’ based on the available NSAIDs and corresponding codes presented in Supplementary Information 3.

Depression status

Depression status at baseline was defined as self-reported depression (Field 20002, codes 1286 and 1531), antidepressants medication use (Field 20003, medication codes shown in Supplementary Information 4), history of major depression derived by responses to mental health

questions on the touchscreen questionnaire (Field 20126, codes 3, 4 or 5), or baseline hospital inpatient records with the International Classification of Diseases version10 (ICD-10) codes F32 and F33 in main or secondary diagnoses (Fields 41270 and 41280), according to several previous studies [14-17].

Number of diet and anxiety-related comorbidities

We used self-reported medical conditions (Field 20002), medication use (Field 20003), and hospital inpatient admission records (Fields 41270 and 41280) based on the ICD-10 codes to ascertain prevalent diet and anxiety-related comorbidities for each individual at baseline (yes or no). In this analysis, we defined diet and anxiety-related comorbidities as 7 diseases including cardiovascular diseases, hypertension, hyperlipidemia, type-2 diabetes mellitus, cancers, digestive diseases, and chronic kidney diseases based on previous research [18-22]. We referred to previous studies for the specific UK Biobank field codes in the study [23-29], and presented this information in the Supplemental Information 7. The number of diet and anxiety-related comorbidities was the sum of the seven diseases an individual was diagnosed at baseline.

Supplementary Information S3. Nonsteroidal anti-inflammatory drugs (NSAIDs) and corresponding drug IDs in the UK Biobank

**Drug ID**	**Drug Name**
1140925806	Aceclofenac
1140868226	Aspirin
1141176662	Celecoxib
1141164746	Dexketoprofen
1140878036	Diclofenac Sodium
1140871188	Etodolac
1141180140	Etoricoxib
1140871226	Fenoprofen
1140871310	Ibuprofen
1140871506	Ketoprofen
1140871542	Mefenamic Acid
1140926732	Meloxicam
1140875336	Nabumetone
1140871462	Naproxen
1140871666	Piroxicam
1140871604	Sulindac
1140875346	Tenoxicam
1140871614	Tiaprofenic Acid

Supplementary Information S4. The code list of antidepressants in the UK Biobank

**Drug ID**	**Drug Name**
1140879616	amitriptyline
1140921600	citalopram
1140879540	fluoxetine
1140867878	sertraline

1140916282	venlafaxine
1140909806	dosulepin
1140867888	paroxetine
1141152732	mirtazapine
1141180212	escitalopram
1140879634	trazodone
1140867876	prozac
1140882236	seroxat
1141190158	cipralex
1141200564	duloxetine
1140867726	lofepramine
1140879620	clomipramine
1140867818	nortriptyline
1140879630	imipramine
1140879628	dothiepin
1141151946	cipramil
1140867948	amitriptyline
1140867624	prothiaden
1140867756	trimipramine
1140867884	lustral
1141151978	reboxetine
1141152736	zispin
1141201834	cymbalta
1140867690	anafranil
1140867640	doxepin
1140867920	moclobemide
1140867850	phenelzine
1140879544	fluvoxamine
1141200570	yentreve
1140867934	triptafen
1140867758	surmontil
1140867914	tranylcypromine
1140867820	allegron
1141151982	edronax
1140882244	molipaxin
1140879556	mianserin
1140867852	nardil
1140867860	faverin
1140917460	nefazodone
1140867938	amitriptyline+chlordiazepoxide
1140867856	isocarboxazid
1140867922	manerix
1140910820	maoi
1140882312	sinequan

1140867944	tranylcypromine+trifluoperazine
1140867784	ludiomil
1140867812	norval
1140867668	tryptizol

Supplementary Information S5. The list of excluded anxiety-related disorders at baseline in the sensitivity analysis

Names of conditions[30]
Bipolar affective disorder
Brain cancer/primary malignant tumour
Brain haemorrhage
Brain/intracranial abscess
Cerebral aneurysm
Cerebral palsy
Chronic/degenerative neurological problem
Dementia/Alzheimer's disease/cognitive impairment
Encephalitis
Epilepsy
Head injury
Infection of nervous system
Ischaemic stroke
Meningeal cancer/malignant meningioma
Meningioma (benign)
Meningitis
Motor neurone disease
Multiple sclerosis
Neurological injury/trauma
Neuroma (benign)
Other demyelinating condition
Other neurological problem
Parkinson's disease
Psychotic disorders (ICD-10 F20-F29)
Spina bifida
Stroke
Subarachnoid haemorrhage
Subdural haematoma
Transient ischaemic attack
Field odes for medical conditions as listed above
Field 6150 = 3
Field 20001 = 1031, 1032
Field 20002 = 1082 1083 1086 1524 1262 1397 1683 1245 1246 1491 1425 1433 1258 1263 1264 1266 1244 1583 1659 1259 1240 1434
Fields 41202 and 41204 = F20 – F29, F31

Supplementary Information S6. Calculation of pure alcohol intake in the UK Biobank

	**Red wine (glasses)** *Pure alcohol: 10.7g/100ml*	**White wine (glasses)** *Pure alcohol: 9.7g/100ml*	**Beer/cider (pints)** *Pure alcohol: 3.5g/100ml*	**Spirits (measures)** *Pure alcohol: 31.7g/100ml*	**Fortified wine (glasses)** *Pure alcohol: 14.4g/100ml*	**Others (glasses)** *Pure alcohol: 3.5g/100ml*
**Intake frequency (Field 1558)**						
**Never**	/	/	/	/	/	/
**Special occasions only**	Field 4407; 1 glass = 125ml	Field 4418; 1 glass = 125ml	Field 4429; 1 pint = 473.2 ml	Field 4440; 1 measure = 28 ml	Field 4451; 1 glass = 125ml	Field 4462; 1 glass = 125ml
**1-3 times/month**						
**1-2 times/week**	Field 1568; 1 glass = 125ml	Field 1578; 1 glass = 125ml	Field 1588; 1 pint = 473.2 ml	Field 1598; 1 measure = 28 ml	Field 1608; 1 glass = 125ml	Field 5364; 1 glass = 125ml
**3-4 times/week**						
**5-7 times/week**						

Supplementary Information S7. Codes used in the UK Biobank to define prevalent comorbidities at baseline

**Prevalent diseases at baseline**	**UKB field IDs and codes for self-reported conditions**	**ICD-9**	**ICD-10**	**OPCS-4**
Cancer [23]	20001	140-208	C00-C97	
Cardiovascular Disease [23]	6150 (1,2,3), 20002 (1074,1075,1081,1583,1086, 1491)	410-414, 430-434, 436	I20-I25, I60-I64	
Hypertension [23]	6150 (4), 20002 (1065,1072), 6153 (2), 6177 (2)	401-405	I10-I13, I15, O10	
Hyperlipidemia [23]	20002 (1473), 6153 (1), 6177 (1)	272	E78	
Type-2 diabetes mellitus [24]	2443(1), 2976, 6153(3), 6177(3), 20002(1220, 1222, 1223)	250	E10-E14	

Chronic kidney disease [25-28]	20002 (1427 ,1607, 1192, 1193, 1194, 1519, 1520)	75315, 584, 585, 586, 587, 588;	I12.0, I13.1, I13.2, N028, N03, N06, N08, N11, N12, N13, N14, N15, N16, N17, N18, N19, N20, N21, E112, E85.3, Q60.1, Q611, Q612, Q613, T82.4, T86.1, Y60.2, Y61.2, Y62.2, Y84.1, Z49.0, Z49.1, Z49.2, Z94.0, Z99.2	M01, M01.1, M01.2, M01.3, M01.4, M01.5, M01.8, M01.9, M02.3, M08.4, M17.2, M17.4, M17.8, M17.9, X40.1, X40.2, X40.3, X40.4, X40.5, X40.6, X40.7, X40.8, X40.9, X41.1, X41.2, X41.8, X41.9, X42.1, X42.8, X42.9, X43.1
Digestive disease [29]	20002 (1134, 1135, 1136, 1137, 1138, 1139, 1140, 1141, 1142, 1143, 1154, 1155, 1156, 1157, 1158, 1159, 1160, 1161, 1162, 1163, 1164, 1165, 1190, 1191, 1400, 1432, 1456, 1457, 1458, 1459, 1460, 1461, 1462, 1463, 1475, 1501, 1503, 1504, 1505, 1506, 1509, 1510, 1511, 1512, 1513, 1562, 1599, 1600, 1601, 1602, 1603, 1604)	520-579	K00-K93	

Abbreviations: ICD, International Classification of Diseases; OPCS, Office of Population, Censuses and Surveys: Classification of interventions and Procedures; UKB: UK Biobank

Supplemental Table S1. Stratified associations between E-DII scores and risk of total anxiety disorders in the UK Biobank by sociodemographic and comorbidity factors (n=96679)

	Quartile 1	Quartile 2	Quartile 3	Quartile 4	P_{trend}[a]	$HR_{continuous}$ (95%CI)[b]	$P_{interaction}$[c]
**Age group[d]**							0.66
<58 years	Ref.	1.03 (0.88-1.21)	1.02 (0.86-1.20)	1.14 (0.97-1.34)	0.10	1.05 (0.99-1.11)	
≥58 years	Ref.	0.97 (0.85-1.12)	0.97 (0.84-1.12)	1.11 (0.95-1.29)	0.24	1.03 (0.98-1.09)	
**Education qualification[e]**							0.75
College or university degree/vocational qualification	Ref.	1.02 (0.90-1.16)	1.02 (0.89-1.16)	1.17 (1.03-1.34)	0.01	1.07 (1.01-1.12)	
National examination at age 17-18	Ref.	0.95 (0.61-1.48)	1.02 (0.66-1.58)	1.24 (0.80-1.91)	0.56	1.05 (0.89-1.23)	
National examination at age 16	Ref.	0.93 (0.71-1.21)	0.98 (0.75-1.29)	1.03 (0.79-1.35)	0.95	1.00 (0.90-1.10)	
**Townsend deprivation index[f]**							0.49
Least deprived	Ref.	0.96 (0.80-1.16)	0.93 (0.76-1.13)	1.05 (0.86-1.28)	0.74	1.01 (0.94-1.09)	
Intermediate	Ref.	0.87 (0.72-1.05)	0.99 (0.83-1.20)	0.99 (0.81-1.20)	0.82	0.99 (0.93-1.06)	
Most deprived	Ref.	1.15 (0.96-1.38)	1.03 (0.85-1.24)	1.32 (1.10-1.57)	0.002	1.11 (1.04-1.19)	
**Number of related comorbidities[g]**							0.42
0	Ref.	0.98 (0.82-1.18)	1.00 (0.84-1.20)	1.03 (0.85-1.25)	0.34	1.03 (0.97-1.11)	
1-2	Ref.	0.98 (0.84-1.13)	0.93 (0.80-1.08)	1.06 (0.91-1.24)	0.53	1.02 (0.96-1.08)	
≥3	Ref.	1.11 (0.84-1.48)	1.18 (0.88-1.57)	1.61 (1.21-2.12)	0.01	1.15 (1.04-1.27)	

Abbreviations: BMI, body mass index; E-DII, energy-adjusted dietary inflammatory index; GCSE, General Certificate of Secondary Education; HR, hazard ratio.

[a] P_{trend} was calculated using the continuous E-DII score in the multivariable-adjusted COX model with adjustment of age group, sex, average total energy (kcal/day), ethnicity, education qualification, Townsend deprivation index, cigarette smoking status, alcohol drinking status, physical activity, BMI status, supplement use, depression status, sleep quality, number of related comorbidities which were all treated same as those in Table 1.

[b] The continuous HR and 95%CI were calculated in association with one standard deviation increase in E-DII score.

[c] P-interaction was calculated by adding the cross-product of each effect modifier in this table and E-DII quartiles in the multivariable adjusted COX model as stated in a.

[d] Age group was divided to two groups based on the median of the distribution.

[e] National examination at age 17-18 refers to the intermediate qualifications including ‘A levels/AS levels or equivalent’, and National examination at age 16 refers to the lowest qualifications including ‘O levels/GCSEs or equivalent’ and ‘CSEs or equivalent’.

[f] Townsend deprivation index was categorized to tertiles with cut-offs shown in Table 1, with larger values indicating areas with a higher level of deprivation.

[g] The related comorbidities were seven chronic diseases including cardiovascular diseases, hypertension, hyperlipidemia, Type-2 diabetes mellitus, cancers, digestive diseases and chronic kidney diseases. The definition in the UK Biobank for each disease was detailed in Supplemental Method section.

Supplemental Table S2. Stratified associations between E-DII scores and risk of phobic anxiety disorders in the UK Biobank by sociodemographic and comorbidity factors (n=96679)

	Quartile 1	Quartile 2	Quartile 3	Quartile 4	P_{trend}[a]	$HR_{continuous}$ (95%CI)[b]	$P_{interaction}$[c]
**Age group[d]**							0.59
<58 years	Ref.	0.89 (0.52-1.51)	0.89 (0.52-1.50)	1.05 (0.63-1.75)	0.48	1.07 (0.89-1.29)	
≥58 years	Ref.	1.29 (0.88-1.89)	0.87 (0.56-1.36)	1.29 (0.84-1.97)	0.22	1.10 (0.94-1.28)	
**Education qualification[e]**							0.69
College or university degree/vocational qualification	Ref.	1.19 (0.83-1.71)	0.92 (0.62-1.38)	1.24 (0.84-1.84)	0.20	1.10 (0.95-1.27)	
National examination at age 17-18	Ref.	3.76 (0.80-17.81)	1.77 (0.34-9.40)	1.62 (0.31-8.59)	0.71	0.92 (0.58-1.44)	
National examination at age 16	Ref.	0.77 (0.34-1.73)	0.45 (0.17-1.18)	0.90 (0.40-2.03)	0.98	1.01 (0.73-1.39)	
**Townsend deprivation index[f]**							0.60
Least deprived	Ref.	0.94 (0.54-1.64)	0.43 (0.21-0.89)	0.84 (0.44-1.58)	0.31	0.89 (0.70-1.12)	
Intermediate	Ref.	0.84 (0.49-1.43)	0.99 (0.58-1.68)	1.10 (0.64-1.88)	0.42	1.09 (0.89-1.33)	
Most deprived	Ref.	1.69 (1.00-2.85)	1.24 (0.70-2.19)	1.67 (0.97-2.90)	0.03	1.24 (1.02-1.50)	
**Number of related comorbidities[g]**							0.09
0	Ref.	1.70 (1.01-2.88)	1.29 (0.74-2.27)	1.24 (0.68-2.24)	0.66	1.05 (0.86-1.27)	
1-2	Ref.	0.90 (0.57-1.42)	0.69 (0.42-1.14)	0.88 (0.54-1.43)	0.98	1.00 (0.84-1.20)	
≥3	Ref.	0.87 (0.41-1.87)	0.75 (0.32-1.73)	2.03 (1.02-4.04)	0.01	1.41 (1.07-1.85)	

Abbreviations: BMI, body mass index; E-DII, energy-adjusted dietary inflammatory index; GCSE, General Certificate of Secondary Education; HR, hazard ratio.
[a] P_{trend} was calculated using the continuous E-DII score in the multivariable-adjusted COX model with adjustment of age group, sex, average total energy (kcal/day), ethnicity, education qualification, Townsend deprivation index, cigarette smoking status, alcohol drinking status, depression status, sleep quality, physical activity, BMI status, supplement use, number of related comorbidities which were all treated same as those in Table 1.
[b] The continuous HR and 95%CI were calculated in association with one standard deviation increase in E-DII score.
[c] P-interaction was calculated by adding the cross-product of each effect modifier in this table and E-DII quartiles in the multivariable adjusted COX model as stated in a.
[d] Age group was divided to two groups based on the median of the distribution.

[e] National examination at age 17-18 refers to the intermediate qualifications including 'A levels/AS levels or equivalent', and National examination at age 16 refers to the lowest qualifications including 'O levels/GCSEs or equivalent' and 'CSEs or equivalent'.
[f] Townsend deprivation index was categorized to tertiles with cut-offs shown in Table 1, with larger values indicating areas with a higher level of deprivation.
[g] The related comorbidities were seven chronic diseases including cardiovascular diseases, hypertension, hyperlipidemia, Type-2 diabetes mellitus, cancers, digestive diseases and chronic kidney diseases. The definition in the UK Biobank for each disease was detailed in Supplemental Method section.

Supplemental Table S3. Stratified associations between E-DII scores and risk of other anxiety disorders in the UK Biobank by sociodemographic and comorbidity factors (n=96679)

	Quartile 1	Quartile 2	Quartile 3	Quartile 4	P_{trend}[a]	$HR_{continuous}$ (95%CI)[b]	$P_{interaction}$[c]
**Age group[d]**							0.48
<58 years	Ref.	1.06 (0.89-1.25)	1.03 (0.87-1.22)	1.15 (0.97-1.36)	0.13	1.05 (0.99-1.11)	
≥58 years	Ref.	0.92 (0.79-1.07)	0.98 (0.85-1.14)	1.08 (0.92-1.27)	0.38	1.03 (0.97-1.09)	
**Education qualification[e]**							0.69
College or university degree/vocational qualification	Ref.	1.00 (0.87-1.14)	1.02 (0.89-1.17)	1.15 (1.00-1.32)	0.04	1.06 (1.00-1.11)	
National examination at age 17-18	Ref.	0.86 (0.54-1.37)	1.06 (0.67-1.65)	1.25 (0.80-1.95)	0.39	1.08 (0.91-1.27)	
National examination at age 16	Ref.	0.92 (0.70-1.22)	1.03 (0.78-1.36)	1.06 (0.80-1.41)	0.92	1.01 (0.91-1.11)	
**Townsend deprivation index[f]**							0.57
Least deprived	Ref.	0.98 (0.80-1.19)	0.98 (0.80-1.20)	1.06 (0.86-1.31)	0.59	1.02 (0.95-1.10)	
Intermediate	Ref.	0.85 (0.70-1.03)	1.00 (0.82-1.22)	0.98 (0.79-1.20)	0.67	0.98 (0.92-1.06)	
Most deprived	Ref.	1.10 (0.91-1.33)	1.01 (0.83-1.23)	1.27 (1.06-1.54)	0.01	1.10 (1.03-1.18)	
**Number of related comorbidities[g]**							0.55
0	Ref.	0.92 (0.76-1.12)	0.99 (0.82-1.20)	1.02 (0.84-1.24)	0.33	1.04 (0.97-1.11)	
1-2	Ref.	0.97 (0.84-1.14)	0.95 (0.81-1.11)	1.06 (0.91-1.25)	0.66	1.01 (0.96-1.07)	
≥3	Ref.	1.13 (0.84-1.52)	1.24 (0.92-1.68)	1.55 (1.15-2.09)	0.03	1.13 (1.01-1.25)	

Abbreviations: BMI, body mass index; E-DII, energy-adjusted dietary inflammatory index; GCSE, General Certificate of Secondary Education; HR, hazard ratio.
[a] P_{trend} was calculated using the continuous E-DII score in the multivariable-adjusted COX model with adjustment of age group, sex, average total energy (kcal/day), ethnicity, education qualification, Townsend deprivation index, cigarette smoking status, alcohol drinking status, physical activity, BMI status, supplement use, depression status, sleep quality, number of related

comorbidities which were all treated same as those in Table 1.
[b] The continuous HR and 95%CI were calculated in association with one standard deviation increase in E-DII score.
[c] P-interaction was calculated by adding the cross-product of each effect modifier in this table and E-DII quartiles in the multivariable adjusted COX model as stated in a.
[d] Age group was divided to two groups based on the median of the distribution.
[e] National examination at age 17-18 refers to the intermediate qualifications including ‘A levels/AS levels or equivalent’, and National examination at age 16 refers to the lowest qualifications including ‘O levels/GCSEs or equivalent’ and ‘CSEs or equivalent’.
[f] Townsend deprivation index was categorized to tertiles with cut-offs shown in Table 1, with larger values indicating areas with a higher level of deprivation.
[g] The related comorbidities were seven chronic diseases including cardiovascular diseases, hypertension, hyperlipidemia, Type-2 diabetes mellitus, cancers, digestive diseases and chronic kidney diseases. The definition in the UK Biobank for each disease was detailed in Supplemental Method section.

Supplemental Table S4. Multivariable-adjusted associations between baseline inflammation-related lifestyle factors and risk of anxiety outcomes in the UK Biobank (n=96679)

	Total anxiety disorders[a]	Phobic anxiety disorders[a]	Other anxiety disorders[a]
	MV-adjusted HR (95% CI)[b]	MV-adjusted HR (95% CI)[b]	MV-adjusted HR (95% CI)[b]
**BMI status**			
Normal weight, 18.5-24.9 kg/m^2	Ref.	Ref.	Ref.
Underweight, <18.5 kg/m^2	1.50 (1.00-2.26)	1.20 (0.30-4.85)	1.45 (0.94-2.23)
Overweight, 25.0-29.9 kg/m^2	0.98 (0.90-1.07)	1.02 (0.78-1.34)	1.00 (0.91-1.09)
Obese, ≥30 kg/m^2	1.08 (0.98-1.20)	1.40 (1.04-1.89)	1.05 (0.95-1.18)
**Smoking status[c]**			
Never smokers	Ref.	Ref.	Ref.
Past smokers	1.30 (1.20-1.41)	1.02 (0.79-1.31)	1.33 (1.22-1.45)
Current smokers	1.73 (1.51-1.98)	1.57 (1.05-2.35)	1.76 (1.53-2.03)
**Alcohol status[c]**			
Never drinkers	Ref.	Ref.	Ref.
Past drinkers	1.27 (0.99-1.62)	0.62 (0.30-1.28)	1.34 (1.04-1.74)
Current drinkers	0.70 (0.58-0.84)	0.53 (0.33-0.84)	0.73 (0.59-0.89)
**Sleep quality[c, d]**			
Healthy	Ref.	Ref.	Ref.
Intermediate	1.31 (1.20-1.43)	1.33 (1.01-1.74)	1.32 (1.20-1.44)
Poor	1.73 (1.42-2.10)	1.36 (0.72-2.57)	1.75 (1.43-2.15)
**Physical activity level[c]**			
High	Ref.	Ref.	Ref.
Medium	0.96 (0.87-1.07)	0.92 (0.66-1.28)	0.97 (0.87-1.08)
Low	1.08 (0.98-1.20)	1.33 (0.98-1.80)	1.07 (0.97-1.19)

NSAIDs use[d]			
Yes	Ref.	Ref.	Ref.
No	0.82 (0.75-0.89)	1.01 (0.77-1.31)	0.81 (0.74-0.88)
**Vitamin/mineral supplement use[d]**			
Yes	Ref.	Ref.	Ref.
No	0.97 (0.90-1.05)	0.84 (0.67-1.05)	0.97 (0.90-1.06)

Abbreviations: BMI, body mass index; E-DII, energy-adjusted dietary inflammatory index; HR, hazard ratio; MV, multivariable; NSAIDs, nonsteroidal anti-inflammatory drugs
[a] Total anxiety disorders include phobic anxiety disorders (ICD-10 F40), other anxiety disorders (ICD-10 F41) and obsessive-compulsive disorder (ICD-10 F42).
[b] The MV-adjusted COX model was adjusted for age group, sex, average total energy (kcal/day), E-DII quartiles, ethnicity, education qualification, Townsend deprivation index, depression status, sleep quality, cigarette smoking status, alcohol drinking status, physical activity level, BMI status, supplement use, number of related comorbidities at baseline with all these covariates defined same as those listed in Table 1.
[c] The "unknown" category was not included for these lifestyle factors.
[d] These inflammation-related lifestyles were categorized and defined same as those listed in Table 1.

Supplemental Table S5. Joint effects of binary E-DII groups and inflammation-related lifestyle factors on the risk of phobic and other anxiety disorders in the UK Biobank (n=96679)

	E-DII groups[a]	Phobic anxiety disorders[b]				Other anxiety disorders[c]			
		Cases No. /person-years	MV-adjusted HR (95% CI)[d]	Additive interaction[e]	Multiplicative interaction[f]	Cases No. /person-years	MV-adjusted HR (95% CI)[d]	Additive interaction[e]	Multiplicative interaction[f]
**BMI status[g, h]**									
Normal	Low	64/193479	Ref.			518/191554	Ref.		
Normal	High	46/165467	0.90 (0.61-1.32)			449/163859	1.06 (0.93-1.20)		
Underweight	Low	2/2625	1.94 (0.48-7.95)			10/2580	1.22 (0.65-2.28)		
Underweight	High	0/1785	NA	NA	NA	11/1738	1.86 (1.02-3.39)	0.59 (P=0.39)	0.57 (P=0.40)
Overweight	Low	59/175962	1.01 (0.71-1.44)			473/174285	1.00 (0.88-1.13)		
Overweight	High	53/188712	0.94 (0.64-1.37)	0.03 (P=0.90)	0.03 (P=0.90)	494/187068	1.05 (0.93-1.20)	-0.01 (P=0.97)	-0.01 (P=0.97)
Obese	Low	39/76952	1.24 (0.82-1.87)			260/76062	1.05 (0.90-1.22)		
Obese	High	48/91481	1.43 (0.96-2.13)	0.29 (P=0.38)	0.31 (P=0.39)	311/90522	1.13 (0.97-1.31)	0.02 (P=0.87)	0.02 (P=0.89)
**Cigarette smoking status[g]**									

Never	Low	101/266256	Ref.			661/263830	Ref.		
Never	High	70/252003	0.81 (0.60-1.11)			603/249958	1.05 (0.94-1.18)		
Past smokers	Low	53/160732	0.85 (0.60-1.19)			518/158933	1.32 (1.18-1.49)		
Past smokers	High	58/157757	1.03 (0.73-1.44)	0.37 (P=0.08)	0.34 (P=0.10)	497/156101	1.40 (1.24-1.58)	0.03 (P=0.79)	0.01 (P=0.92)
Current smokers	Low	10/21124	1.31 (0.68-2.52)			77/20829	1.55 (1.22-1.96)		
Current smokers	High	19/36800	1.56 (0.94-2.59)	0.44 (P=0.43)	0.50 (P=0.36)	164/36249	2.01 (1.69-2.40)	0.42 (P=0.09)	0.38 (P=0.15)
**Alcohol drinking status[g]**									
Never	Low	11/13491	Ref.			46/13312	Ref.		
Never	High	9/12721	0.92 (0.38-2.22)			54/12542	1.30 (0.88-1.92)		
Past drinkers	Low	6/13242	0.57 (0.21-1.56)			66/12993	1.34 (0.92-1.96)		
Past drinkers	High	6/11816	0.62 (0.23-1.71)	0.14 (P=0.80)	0.10 (P=0.81)	76/11568	1.74 (1.20-2.52)	0.10 (P=0.77)	-0.002 (P=0.99)
Current drinkers	Low	146/422077	0.51 (0.27-0.95)			1148/417969	0.81 (0.60-1.09)		
Current drinkers	High	132/422631	0.50 (0.26-0.93)	0.07 (P=0.87)	0.03 (P=0.90)	1134/418802	0.85 (0.63-1.14)	-0.26 (P=0.31)	-0.20 (P=0.29)
**Sleep quality[i]**									
Healthy	Low	47/167576	Ref.			387/166137	Ref.		
Healthy	High	36/148729	0.93 (0.60-1.45)			325/147624	1.02 (0.87-1.18)		
Poor	Low	5/10462	1.36 (0.54-3.44)			49/10252	1.88 (1.39-2.53)		
Poor	High	6/14549	1.30 (0.55-3.07)	0.001(P=0.99)	0.04 (P=0.98)	60/14308	1.71 (1.30-2.26)	-0.18 (P=0.61)	-0.21 (P=0.60)
**Physical activity level[i]**									
High	Low	36/139052	Ref.			368/137583	Ref.		
High	High	37/118318	1.35 (0.85-2.15)			321/117269	1.10 (0.94-1.28)		
Low	Low	50/115203	1.57 (1.02-2.42)			353/114039	1.11 (0.96-1.29)		
Low	High	53/141310	1.50 (0.98-2.31)	-0.42 (P=0.34)	-0.62 (P=0.26)	413/139978	1.14 (0.99-1.32)	-0.07 (P=0.56)	-0.08 (P=0.52)
**NSAIDs use**									
Yes	Low	40/100118	Ref.			362/98871	Ref.		

Yes	High	42/104099	1.05 (0.68-1.63)			369/102843	1.03 (0.89-1.19)		
No	Low	124/348900	1.07 (0.74-1.54)			899/345609	0.79 (0.70-0.89)		
No	High	105/343346	1.00 (0.68-1.45)	-0.13 (P=0.65)	-0.12 (P=0.64)	896/340344	0.85 (0.75-0.96)	0.03 (P=0.69)	0.04 (P=0.59)
**Vitamin/mineral supplement use**									
Yes	Low	78/201680	Ref.			604/199532	Ref.		
Yes	High	62/151725	1.10 (0.78-1.55)			434/150378	1.00 (0.88-1.13)		
No	Low	86/247338	0.95 (0.69-1.29)			657/244948	0.93 (0.83-1.04)		
No	High	85/295719	0.82 (0.59-1.13)	-0.23 (P=0.34)	-0.23 (P=0.29)	831/292808	1.03 (0.92-1.15)	0.11 (P=0.18)	0.10 (P=0.18)

Abbreviations: BMI, body mass index; E-DII, energy-adjusted dietary inflammatory index; HR, hazard ratio; MV, multivariable; NSAIDs, nonsteroidal anti-inflammatory drugs; OCD, obsessive-compulsive disorder; RERI, relative excess risk due to interaction

[a] E-DII was divided into two groups using the median (-0.03) as the cutoff in the joint effect analyses with the high E-DII group as the risk group for diet-associated inflammation while the low E-DII group as the reference level.

[b] Phobic anxiety disorders (ICD-10 F40) included agoraphobia, social phobias, specific phobias, other phobic anxiety disorders, and unspecified phobic anxiety disorder.

[c] Other anxiety disorders (ICD-10 F41) included panic disorder, generalized anxiety disorder, mixed anxiety and depressive disorder, other mixed anxiety disorders, other specified anxiety disorders, unspecified anxiety disorder

[d] The MV-adjusted COX model was adjusted for age group, average total energy (kcal/day), E-DII (low, high), ethnicity, education qualification, Townsend deprivation index, cigarette smoking status, alcohol drinking status, physical activity level, BMI status, supplement use, depression status, sleep quality, number of related comorbidities at baseline with all these variables treated same as those listed in Table 1.HRs and 95% CIs of incident anxiety outcomes for joint group of the binary E-DII joined with a baseline inflammation-related lifestyle were calculated when treating subjects at the low E-DII group and the most anti-inflammatory level of the lifestyle as the referent.

[e] RERI was calculated to estimate the additive interaction based on the Rothman's method with P value reported. In the additive interaction, the risk group for each lifestyle was each non-reference group and the risk group for binary E-DII was high E-DII group.

[f] We calculated the multiplicative interaction by obtaining the difference between the observed HR for the joint association (i.e., HR among participants having both risk factors versus those who had neither factor) and the expected HR for the joint effect of two independent risk factors (i.e., the product of two adjusted HRs each from the group of only having one risk factor) based on the joint-effect approach. P-value for multiplicative interaction was calculated with the cross-product of binary E-DII and each inflammation-related lifestyle in the MV-adjusted COX model.

[g] These inflammation-related lifestyles were all nominal variables with more than two levels, therefore, we calculated the additive and multiplicative interaction indicators and corresponding P-values for each group than the reference lifestyle group as a separate independent risk factor.

[h] The underweight group did not have joint effect estimates and 95%CIs for phobic anxiety disorders due to no case

[i] For these two lifestyle variables, we only included the two extreme levels as these are ordinal variables.

Inflammation-related lifestyles	E-DII groups [a]	Cases No. /person-years	Multivariable-adjusted Hazard Ratio (95% Confidence Interval)[b]	Additive interaction (RERI) [c]	Multiplicative interaction ($HR_{observed}$ - $HR_{expected}$) [d]
**BMI status [e]**					
Normal	Low	112/53124	Ref.		
Normal	High	143/70898	0.91 (0.71-1.17)		
Underweight	Low	0/58	NA		
Underweight	High	1/129	2.73 (0.38-19.64)	NA	NA
Overweight	Low	160/77412	0.89 (0.70-1.14)		
Overweight	High	253/117949	0.90 (0.72-1.12)	0.09	0.09
Obese	Low	75/28995	0.95 (0.70-1.28)		
Obese	High	141/51187	0.98 (0.75-1.26)	0.12	0.12
**Cigarette smoking status [e]**					
Never	Low	157/85816	Ref.		
Never	High	228/125087	0.97 (0.79-1.19)		
Past smokers	Low	165/64598	1.34 (1.07-1.68)		
Past smokers	High	236/92299	1.32 (1.07-1.63)	0.01	0.02
Current smokers	Low	24/8798	1.38 (0.89-2.13)		
Current smokers	High	74/22286	1.69 (1.27-2.24)	0.34	0.35
**Alcohol drinking status [e]**					
Never	Low	8/2645	Ref.		
Never	High	20/5001	1.30 (0.57-2.95)		
Past drinkers	Low	17/4643	0.91 (0.39-2.11)		
Past drinkers	High	34/6169	1.28 (0.59-2.77)	0.08	0.10
Current drinkers	Low	321/152267	0.60 (0.29-1.21)		
Current drinkers	High	484/228868	0.57 (0.28-1.16)	-0.32	-0.21
**Sleep quality [f]**					
Healthy	Low	93/55256	Ref.		
Healthy	High	120/74672	0.93 (0.71-1.22)		
Poor	Low	16/4595	1.77 (1.04-3.02)		
Poor	High	28/9282	1.44 (0.94-2.22)	-0.26	-0.21
**Physical activity level [f]**					
High	Low	107/52730	Ref.		
High	High	144/68470	1.00 (0.78-1.29)		
Low	Low	106/41695	1.22 (0.93-1.60)		
Low	High	194/76782	1.17 (0.92-1.49)	-0.04	-0.05
**NSAIDs use**					
Yes	Low	105/40040	Ref.		
Yes	High	179/57440	1.14 (0.90-1.46)		
No	Low	242/119549	0.94 (0.74-1.20)		
No	High	359/182723	0.86 (0.69-1.09)	-0.22	-0.21
**Vitamin/mineral supplement use**					
Yes	Low	155/63909	Ref.		
Yes	High	157/72742	0.85 (0.68-1.07)		
No	Low	192/95681	0.82 (0.66-1.02)		
No	High	381/167421	0.88 (0.73-1.07)	0.21 [g]	0.18

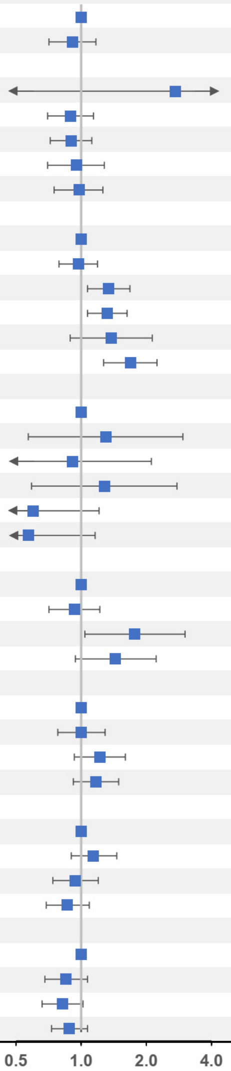

Supplemental Figure S1. Joint effects of binary E-DII groups and inflammation-related lifestyles on the risk of total anxiety disorders among males in the UK Biobank (n=43684)

Abbreviations: BMI, body mass index; E-DII, energy-adjusted dietary inflammatory index; HR, hazard ratio; MV, multivariable; NSAIDs, nonsteroidal anti-inflammatory drugs; RERI, relative excess risk due to interaction. [a] E-DII score was divided into two groups using the median (-0.03) as the cutoff with the high E-DII group as the risk group for diet-associated inflammation while the low E-DII group as the reference level. [b] The multivariable-adjusted Cox model was adjusted for same covariates as those listed in Table 1.We calculated the HRs and 95% CIs of total anxiety disorders for each combined group of the binary E-DII joined with a baseline inflammation-related lifestyle when treating subjects at the low E-DII group and the most anti-inflammatory level of the lifestyle as the referent. [c] RERI was calculated to estimate the additive interaction based on the Rothman's method with P value reported. All P values>0.10 in this figure if not specified. [d] We calculated the multiplicative interaction by obtaining the difference between the observed HR for the joint association and the expected HR for the joint effect of two independent risk factors based on the joint-effect approach. P-value for multiplicative interaction was calculated with the cross-product of binary E-DII and each inflammation-related lifestyle in the multivariable-adjusted Cox model. All P values>0.10 in this figure if not specified. [e] These inflammation-related lifestyles were all nominal variables with more than two levels, therefore, we calculated the additive and multiplicative interaction indicators and corresponding P-values for risk group as a separate independent risk factor. [f] For these two lifestyle variables, we only included the two extreme levels as these are ordinal variables. [g] P value=0.09.

Inflammation-related lifestyles	E-DII groups [a]	Cases No. /person-years	Multivariable-adjusted Hazard Ratio (95% Confidence Interval)[b]	Additive interaction (RERI) [c]	Multiplicative interaction ($HR_{observed}$ - $HR_{expected}$) [d]
**BMI status [e]**					
Normal	Low	465/138140	Ref.		
Normal	High	346/92770	1.07 (0.93-1.24)		
Underweight	Low	12/2516	1.38 (0.78-2.45)		
Underweight	High	11/1608	1.83 (1.00-3.33)	0.37	0.35
Overweight	Low	358/96692	1.00 (0.87-1.15)		
Overweight	High	278/68940	1.06 (0.91-1.23)	-0.01	-0.01
Obese	Low	217/46940	1.08 (0.92-1.28)		
Obese	High	213/39150	1.23 (1.04-1.46)	0.07	0.07
**Cigarette smoking status [e]**					
Never	Low	588/177617	Ref.		
Never	High	433/124603	1.04 (0.91-1.18)		
Past smokers	Low	399/94156	1.25 (1.10-1.42)		
Past smokers	High	309/63591	1.39 (1.21-1.61)	0.11	0.09
Current smokers	Low	61/12004	1.55 (1.19-2.03)		
Current smokers	High	105/13885	2.16 (1.74-2.67)	0.57 [g]	0.55 [h]
**Alcohol drinking status [e]**					
Never	Low	45/10635	Ref.		
Never	High	43/7515	1.25 (0.82-1.90)		
Past drinkers	Low	54/8337	1.35 (0.90-2.01)		
Past drinkers	High	50/5362	1.72 (1.15-2.59)	0.13	0.03
Current drinkers	Low	952/265147	0.83 (0.61-1.12)		
Current drinkers	High	754/189439	0.89 (0.65-1.20)	-0.19	-0.15
**Sleep quality [f]**					
Healthy	Low	338/110686	Ref.		
Healthy	High	234/72803	1.03 (0.87-1.22)		
Poor	Low	37/5648	1.81 (1.28-2.54)		
Poor	High	38/4998	1.89 (1.35-2.66)	0.05	0.03
**Physical activity level [f]**					
High	Low	293/84728	Ref.		
High	High	209/48677	1.20 (1.00-1.44)		
Low	Low	287/72136	1.11 (0.94-1.31)		
Low	High	260/62997	1.13 (0.95-1.33)	-0.19	-0.20
**NSAIDs use**					
Yes	Low	291/58671	Ref.		
Yes	High	226/45234	0.98 (0.83-1.17)		
No	Low	761/225617	0.76 (0.66-0.87)		
No	High	622/157233	0.86 (0.74-0.99)	0.11	0.12
**Vitamin/mineral supplement use**					
Yes	Low	510/135361	Ref.		
Yes	High	324/77406	1.07 (0.93-1.23)		
No	Low	542/148927	0.98 (0.87-1.11)		
No	High	524/125062	1.08 (0.95-1.22)	0.03	0.03

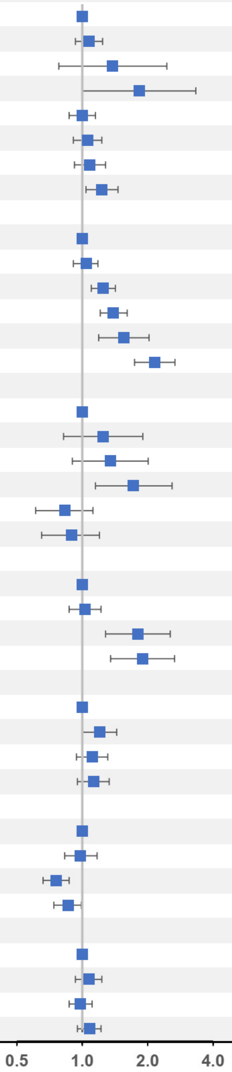

Supplemental Figure S2. Joint effects of binary E-DII groups and inflammation-related lifestyles on the risk of total anxiety disorders among females in the UK Biobank (n=52995)

Abbreviations: BMI, body mass index; E-DII, energy-adjusted dietary inflammatory index; HR, hazard ratio; MV, multivariable; NSAIDs, nonsteroidal anti-inflammatory drugs; RERI, relative excess risk due to interaction. [a] E-DII was divided into two groups using the median (-0.03) as the cutoff with the high E-DII group as the risk group for diet-associated inflammation while the low E-DII group as the reference level. [b] The multivariable-adjusted Cox model was adjusted for same variables as those listed in Table 1. We calculated the HRs and 95% CIs of incident anxiety outcomes for each combined group of the binary E-DII joined with a baseline inflammation-related lifestyle when treating subjects at the low E-DII group and the most anti-inflammatory level of the lifestyle as the referent. [c] RERI was calculated to estimate the additive interaction based on the Rothman's method with P value reported. All P values>0.10 in this figure if not specified. [d] We calculated the multiplicative interaction by obtaining the difference between the observed HR for the joint association and the expected HR for the joint effect of two independent risk factors based on the joint-effect approach. P-value for multiplicative interaction was calculated with the cross-product of binary E-DII and each inflammation-related lifestyle in the multivariable-adjusted Cox model. All P values>0.10 in this figure if not specified. [e] These inflammation-related lifestyles were all nominal variables with more than two levels, therefore, we calculated the additive and multiplicative interaction indicators and corresponding P-values for each risk group as a separate independent risk factor. [f] For these two lifestyle variables, we only included the two extreme levels as these are ordinal variables. [g] P value=0.05. [h] P value=0.09.

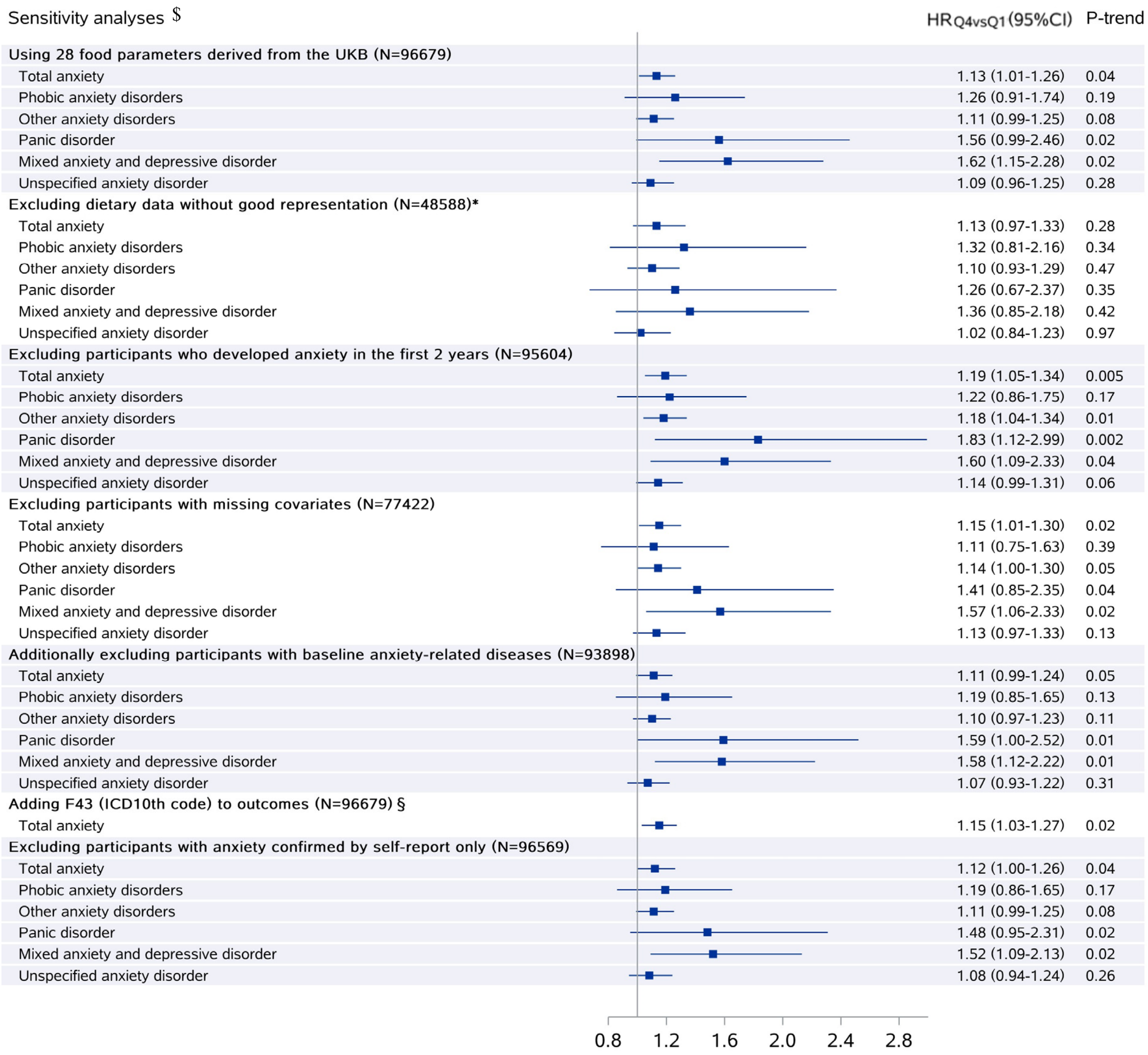

Supplemental Figure S3. Sensitivity analyses of multivariable-adjusted associations between E-DII scores and risk of anxiety outcomes

Abbreviations: E-DII, energy-adjusted dietary inflammatory index; HR, hazard ratio; UKB, UK Biobank; $ The HRs and 95% CIs for each anxiety outcome were calculated by comparing the highest to the lowest quartiles of E-DII score in the multivariable-adjusted Cox regression model with P-trend calculated using the continuous E-DII score in the model. * It referred to only inclusion of participants who had≥2 rounds of typical 24-h dietary recalls comprising at least one weekday and one weekend day diets and whose diet did not have large week-to-week variation (Field 1548). § F43 referred to reaction to severe stress, and adjustment disorders which included PTSD (F43.1).

References

1. Skelton, M.; Rayner, C.; Purves, K.L.; Coleman, J.R.I.; Gaspar, H.A.; Glanville, K.P.; Hunjan, A.K.; Hübel, C.; Breen, G.; Eley, T.C. Self-reported medication use as an alternate phenotyping method for anxiety and depression in the UK Biobank. *Am J Med Genet B Neuropsychiatr Genet* **2021**, *186*, 389-398, doi:10.1002/ajmg.b.32878.
2. Knuppel, A.; Papier, K.; Fensom, G.K.; Appleby, P.N.; Schmidt, J.A.; Tong, T.Y.N.; Travis, R.C.; Key, T.J.; Perez-Cornago, A. Meat intake and cancer risk: prospective analyses in UK Biobank. *International Journal of Epidemiology* **2020**, *49*, 1540-1552, doi:10.1093/ije/dyaa142.
3. Townsend, P.; Phillimore, P.; Beattie, A. Health and deprivation. Inequality and the North.

Revista cubana de higiene y epidemiología **1997**, *35*.
4. Liu, B.; Young, H.; Crowe, F.L.; Benson, V.S.; Spencer, E.A.; Key, T.J.; Appleby, P.N.; Beral, V. Development and evaluation of the Oxford WebQ, a low-cost, web-based method for assessment of previous 24 h dietary intakes in large-scale prospective studies. *Public health nutrition* **2011**, *14*, 1998-2005.
5. Liu, Z.; Suo, C.; Zhao, R.; Yuan, H.; Chen, X. Genetic predisposition, lifestyle risk, and obesity associate with the progression of nonalcoholic fatty liver disease. *Digestive and Liver Disease* **2021**.
6. Bradbury, K.E.; Murphy, N.; Key, T.J. Diet and colorectal cancer in UK Biobank: a prospective study. *International journal of epidemiology* **2020**, *49*, 246-258, doi:10.1093/ije/dyz064.
7. Fan, M.; Sun, D.; Zhou, T.; Heianza, Y.; Lv, J.; Li, L.; Qi, L. Sleep patterns, genetic susceptibility, and incident cardiovascular disease: a prospective study of 385 292 UK biobank participants. *Eur Heart J* **2020**, *41*, 1182-1189, doi:10.1093/eurheartj/ehz849.
8. Hepsomali, P.; Groeger, J.A. Diet, Sleep, and Mental Health: Insights from the UK Biobank Study. *Nutrients* **2021**, *13*, doi:10.3390/nu13082573.
9. Craig, C.L.; Marshall, A.L.; Sjöström, M.; Bauman, A.E.; Booth, M.L.; Ainsworth, B.E.; Pratt, M.; Ekelund, U.; Yngve, A.; Sallis, J.F. International physical activity questionnaire: 12-country reliability and validity. *Medicine & science in sports & exercise* **2003**, *35*, 1381-1395.
10. Cassidy, S.; Chau, J.Y.; Catt, M.; Bauman, A.; Trenell, M.I. Cross-sectional study of diet, physical activity, television viewing and sleep duration in 233 110 adults from the UK Biobank; the behavioural phenotype of cardiovascular disease and type 2 diabetes. *BMJ open* **2016**, *6*, e010038.
11. Committee, I.R. Guidelines for data processing and analysis of the International Physical Activity Questionnaire (IPAQ)-short and long forms. *http://www. ipaq. ki. se/scoring. pdf* **2005**.
12. Seidell, J.C.; Flegal, K.M. Assessing obesity: classification and epidemiology. *British Medical Bulletin* **1997**, *53*, 238-252, doi:10.1093/oxfordjournals.bmb.a011611.
13. Green, H.D.; Beaumont, R.N.; Thomas, A.; Hamilton, B.; Wood, A.R.; Sharp, S.; Jones, S.E.; Tyrrell, J.; Walker, G.; Goodhand, J.; et al. Genome-Wide Association Study of Microscopic Colitis in the UK Biobank Confirms Immune-Related Pathogenesis. *J Crohns Colitis* **2019**, *13*, 1578-1582, doi:10.1093/ecco-jcc/jjz104.
14. Zhang, Z.; Yang, X.; Jia, Y.; Wen, Y.; Cheng, S.; Meng, P.; Li, C.; Zhang, H.; Pan, C.; Zhang, J.; et al. Vitamin D and the Risks of Depression and Anxiety: An Observational Analysis and Genome-Wide Environment Interaction Study. *Nutrients* **2021**, *13*, doi:10.3390/nu13103343.
15. Cheng, B.; Chu, X.; Yang, X.; Wen, Y.; Jia, Y.; Liang, C.; Yao, Y.; Ye, J.; Cheng, S.; Liu, L.; et al. Dietary Habit Is Associated with Depression and Intelligence: An Observational and Genome-Wide Environmental Interaction Analysis in the UK Biobank Cohort. *Nutrients* **2021**, *13*, doi:10.3390/nu13041150.
16. Ronaldson, A.; Arias de la Torre, J.; Gaughran, F.; Bakolis, I.; Hatch, S.L.; Hotopf, M.; Dregan, A. Prospective associations between vitamin D and depression in middle-aged adults: findings from the UK Biobank cohort. *Psychol Med* **2022**, *52*, 1866-1874,

doi:10.1017/s0033291720003657.
17. Davis, K.A.S.; Cullen, B.; Adams, M.; Brailean, A.; Breen, G.; Coleman, J.R.I.; Dregan, A.; Gaspar, H.A.; Hubel, C.; Lee, W.; et al. Indicators of mental disorders in UK Biobank-A comparison of approaches. *Int J Methods Psychiatr Res* **2019**, *28*, e1796, doi:10.1002/mpr.1796.
18. Sánchez-Villegas, A.; Ruíz-Canela, M.; de la Fuente-Arrillaga, C.; Gea, A.; Shivappa, N.; Hébert, J.R.; Martínez-González, M.A. Dietary inflammatory index, cardiometabolic conditions and depression in the Seguimiento Universidad de Navarra cohort study. *Br J Nutr* **2015**, *114*, 1471-1479, doi:10.1017/s0007114515003074.
19. Adjibade, M.; Lemogne, C.; Touvier, M.; Hercberg, S.; Galan, P.; Assmann, K.E.; Julia, C.; Kesse-Guyot, E. The Inflammatory Potential of the Diet is Directly Associated with Incident Depressive Symptoms Among French Adults. *J Nutr* **2019**, *149*, 1198-1207, doi:10.1093/jn/nxz045.
20. Shakya, P.R.; Melaku, Y.A.; Shivappa, N.; Hébert, J.R.; Adams, R.J.; Page, A.J.; Gill, T.K. Dietary inflammatory index (DII®) and the risk of depression symptoms in adults. *Clin Nutr* **2021**, *40*, 3631-3642, doi:10.1016/j.clnu.2020.12.031.
21. Akbaraly, T.; Kerlau, C.; Wyart, M.; Chevallier, N.; Ndiaye, L.; Shivappa, N.; Hébert, J.R.; Kivimäki, M. Dietary inflammatory index and recurrence of depressive symptoms: Results from the Whitehall II Study. *Clin Psychol Sci* **2016**, *4*, 1125-1134, doi:10.1177/2167702616645777.
22. Vermeulen, E.; Brouwer, I.A.; Stronks, K.; Bandinelli, S.; Ferrucci, L.; Visser, M.; Nicolaou, M. Inflammatory dietary patterns and depressive symptoms in Italian older adults. *Brain Behav Immun* **2018**, *67*, 290-298, doi:10.1016/j.bbi.2017.09.005.
23. Han, H.; Cao, Y.; Feng, C.; Zheng, Y.; Dhana, K.; Zhu, S.; Shang, C.; Yuan, C.; Zong, G. Association of a Healthy Lifestyle With All-Cause and Cause-Specific Mortality Among Individuals With Type 2 Diabetes: A Prospective Study in UK Biobank. *Diabetes Care* **2021**, *45*, 319-329, doi:10.2337/dc21-1512.
24. Said, M.A.; Verweij, N.; van der Harst, P. Associations of Combined Genetic and Lifestyle Risks With Incident Cardiovascular Disease and Diabetes in the UK Biobank Study. *JAMA Cardiology* **2018**, *3*, 693-702, doi:10.1001/jamacardio.2018.1717.
25. Shang, X.; Zhang, X.; Huang, Y.; Zhu, Z.; Zhang, X.; Liu, S.; Liu, J.; Tang, S.; Wang, W.; Yu, H.; et al. Temporal trajectories of important diseases in the life course and premature mortality in the UK Biobank. *Bmc Med* **2022**, *20*, 185, doi:10.1186/s12916-022-02384-3.
26. Honigberg, M.C.; Zekavat, S.M.; Pirruccello, J.P.; Natarajan, P.; Vaduganathan, M. Cardiovascular and Kidney Outcomes Across the Glycemic Spectrum: Insights From the UK Biobank. *J Am Coll Cardiol* **2021**, *78*, 453-464, doi:10.1016/j.jacc.2021.05.004.
27. Drozd, M.; Pujades-Rodriguez, M.; Lillie, P.J.; Straw, S.; Morgan, A.W.; Kearney, M.T.; Witte, K.K.; Cubbon, R.M. Non-communicable disease, sociodemographic factors, and risk of death from infection: a UK Biobank observational cohort study. *Lancet Infect Dis* **2021**, *21*, 1184-1191, doi:10.1016/s1473-3099(20)30978-6.
28. Geng, T.; Li, X.; Ma, H.; Heianza, Y.; Qi, L. Adherence to a Healthy Sleep Pattern and Risk of Chronic Kidney Disease: The UK Biobank Study. *Mayo Clin Proc* **2022**, *97*, 68-77, doi:10.1016/j.mayocp.2021.08.028.
29. Knutson, K.L.; von Schantz, M. Associations between chronotype, morbidity and mortality

in the UK Biobank cohort. *Chronobiol Int* **2018**, *35*, 1045-1053, doi:10.1080/07420528.2018.1454458.

30. Sarris, J.; Thomson, R.; Hargraves, F.; Eaton, M.; de Manincor, M.; Veronese, N.; Solmi, M.; Stubbs, B.; Yung, A.R.; Firth, J. Multiple lifestyle factors and depressed mood: a cross-sectional and longitudinal analysis of the UK Biobank (N = 84,860). *Bmc Med* **2020**, *18*, 354, doi:10.1186/s12916-020-01813-5.
